# Self-Organisation, Thermotropic and Lyotropic Properties of Glycolipids Related to their Biological Implications

**DOI:** 10.2174/1874091X01509010049

**Published:** 2015-08-31

**Authors:** Patrick Garidel, Yani Kaconis, Lena Heinbockel, Matthias Wulf, Sven Gerber, Ariane Munk, Volkmar Vill, Klaus Brandenburg

**Affiliations:** 1 Physikalische Chemie, Martin-Luther-Universität Halle/Wittenberg, Mühlpforte 1, D-06108 Halle/Saale, Germany; 2 Forschungszentrum Borstel, Leibniz-Zentrum für Medizin und Biowissenschaften, Parkallee 1-40, D-23845 Borstel, Germany; 3 Institut für Organische Chemie, Universität Hamburg, Martin-Luther-King-Platz 6, D-20146 Hamburg, Germany

**Keywords:** Glycolipids, lipopolysaccharides, lyotropism, structural polymorphism, supramolecular structures, thermotropism

## Abstract

Glycolipids are amphiphilic molecules which bear an oligo- or polysaccharide as hydrophilic head group and hydrocarbon chains in varying numbers and lengths as hydrophobic part. They play an important role in life science as well as in material science. Their biological and physiological functions are quite diverse, ranging from mediators of cell-cell recognition processes, constituents of membrane domains or as membrane-forming units. Glycolipids form an exceptional class of liquid-crystal mesophases due to the fact that their self-organisation obeys more complex rules as compared to classical monophilic liquid-crystals. Like other amphiphiles, the supra-molecular structures formed by glycolipids are driven by their chemical structure; however, the details of this process are still hardly understood. Based on the synthesis of specific glycolipids with a clearly defined chemical structure, e.g., type and length of the sugar head group, acyl chain linkage, substitution pattern, hydrocarbon chain lengths and saturation, combined with a profound physico-chemical characterisation of the formed mesophases, the principles of the organisation in different aggregate structures of the glycolipids can be obtained. The importance of the observed and formed phases and their properties are discussed with respect to their biological and physiological relevance. The presented data describe briefly the strategies used for the synthesis of the used glycolipids. The main focus, however, lies on the thermotropic as well as lyotropic characterisation of the self-organised structures and formed phases based on physico-chemical and biophysical methods linked to their potential biological implications and relevance.

## 1 INTRODUCTION 

**Fig. (1):**
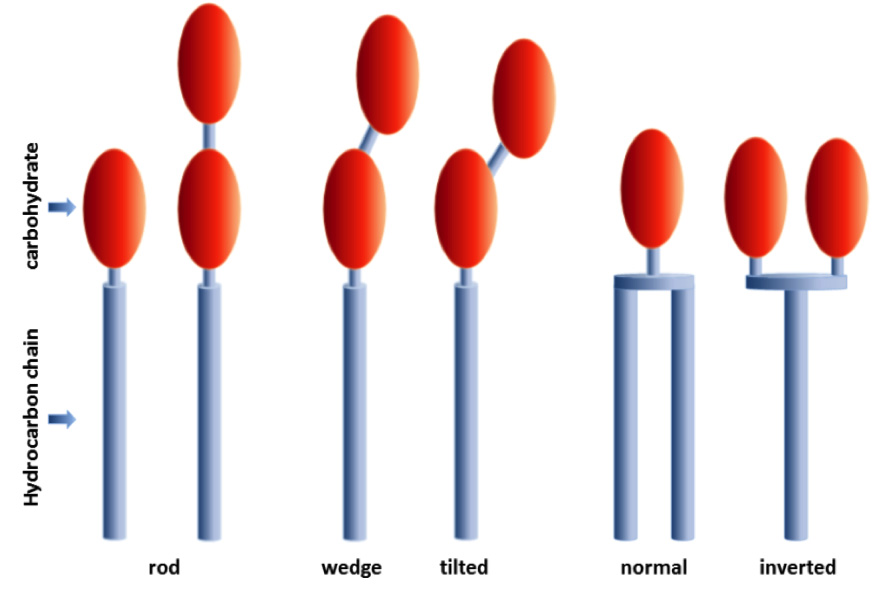
Types of amphiphiles composed of one or more monosaccharide residues (carbohydrate) linked to one ore more hydrocarbon chains.

General speaking, glycolipids are lipids containing carbohydrate. According to the International Union of Pure and Applied Chemistry and International Union of Biochemistry and Molecular Biology, glycolipids are defined as simple derivatives of lipids such as acylglycerols, ceramides, and prenols as well as glycosyl derivatives such as cerebrosides and gangliosides [1, 2]. Glycolipids belong to a larger family of compounds known as glycoconjugates or glycopolymers. The most important classes of glycoconjugates are glycoproteins, glycopeptides, peptidoglycans, proteoglycans, glycolipids and lipopolysaccharides (LPS) [1]. The latter are also denoted as endotoxins [3, 4]. The structures of glycolipids are often extremely complex. This complexity results from the huge variations in the coupled carbohydrate residues, the number and linkage, carbohydrate modifications (e.g. phosphorylation, sulphated), type (e.g. saturated, unsaturated, branched, unbranched hydrocarbon chains) and number of linked hydrocarbon chains (see Fig. **1**). Also the chemical nature of the linker (e.g. ether, ester, amide) allows theformation of a large number of different glycolipid structures. 

Thus, the term glycolipid designates any compound containing one or more monosaccharide residues bound by a glycosidic linkage to a hydrophobic moiety [1]. The term glycoglycerolipid has been defined as a glycolipid containing one or more glycerol residues. 

A number of glycolipids form liquid-crystalline (LC) phases. The liquid-crystalline phase is a state of matter with properties known for a liquid and a solid crystal [5-7]. As an example, the flow property of a liquid-crystal can be similar to a classical liquid; however the orientation of the single molecules is crystal-like. Various types of liquid-crystalline phases are known. They can be identified and characterised using several physico-chemical techniques (see below). For instance, polarisation microscopy is used for the characterisation of liquid-crystalline phases, because the optical properties of the various liquid-crystalline phases are different. Thus, the liquid-crystalline phase is characterised under the polarisation microscope by a very specific and unique texture (see part III of this chapter).

The phases formed by liquid-crystallines are also denoted as a mesomorph state (mesophase). This state describes an organisational state with the molecular order being between the perfect, three-dimensional position as well as orientation ordering of a solid crystal on the one side, and the lack of long-range orientational order as found in isotropic liquids. The terms liquid-crystalline and mesomorph are often used synonymously. 

In general, a liquid-crystalline compound is formed of a rigid moiety and one or more flexible molecule residue. The rigid part aligns the molecules in a specific direction, whereas the flexible parts induce fluidity into the liquid-crystal. The rigid part, i.e. the basic structural unit of a liquid-crystal inducing structural order into the crystals, is referred to as the mesogen unit [1, 5]. The ideal balance of these two parts is essential to form liquid-crystalline materials [8, 9].

Liquid-crystals are often divided into two main groups, namely thermotropes and, lyotropes, which form thermo-tropic, respective lyotropic phases. Thermotropic liquid-crystals exhibit a phase transition into the liquid-crystalline state as temperature is raised [7]. Lyotropic liquid-crystals exhibit phase transitions as a function of both temperature and concentration of the liquid-crystal molecules in a solvent, which is typically water for biological molecules. 

The term amphiphile denotes molecules comprising a polar (hydrophilic) head-group (which may be ionic or non-ionic) attached to a hydrophobic moiety. Typical hydro-phobic groups are saturated or unsaturated hydrocarbon chains. Examples of amphiphilic compounds are the salts of fatty acids, phospholipids or glycolipids. Many amphiphiles are used as detergents. 

**Fig. (2):**
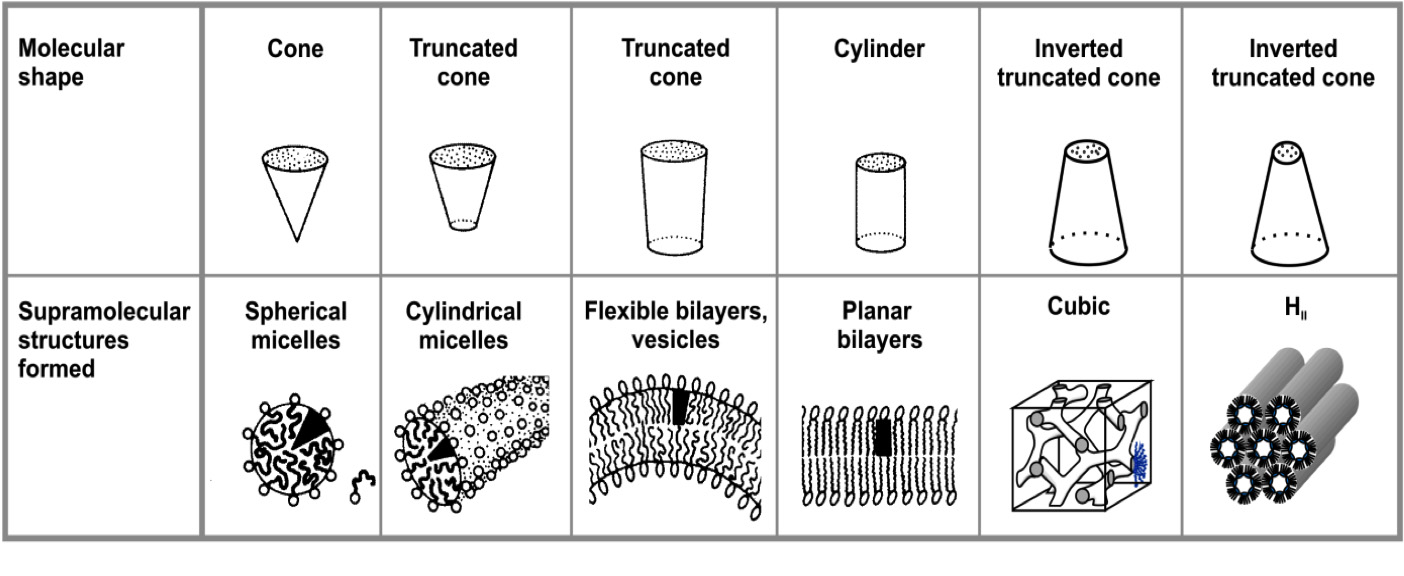
Molecular relationship of amphiphiles related to their supramolecular structure (adapted from Israelachvili (1991) [13]).

**Fig. (3):**
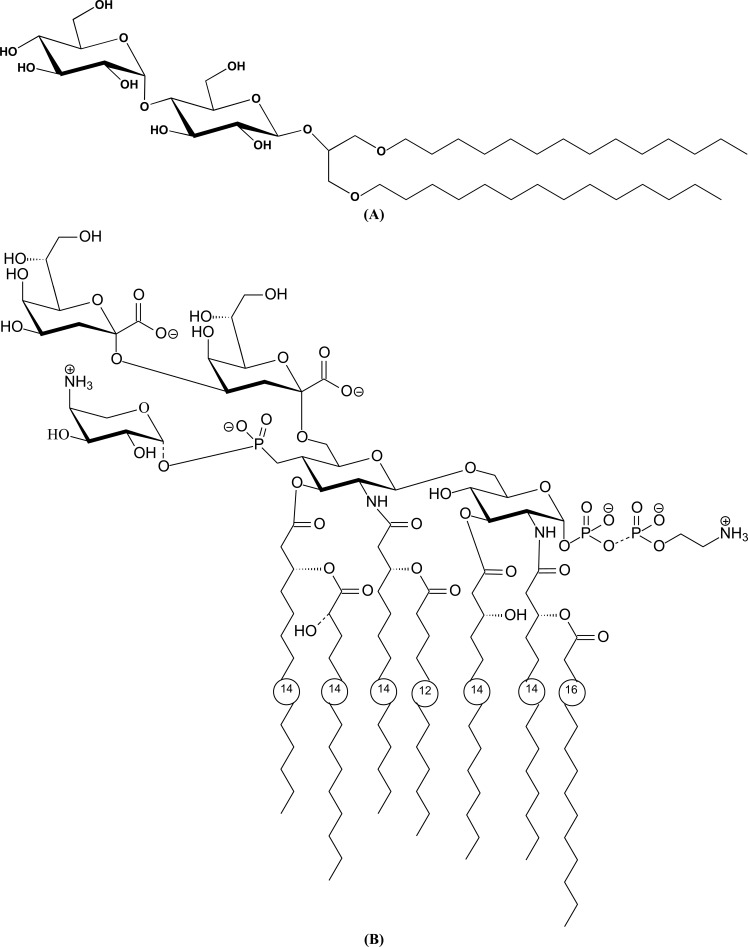
(A). Chemical structure of a glycolipid with a maltose headgroup: 1,3-Di-O-tetradecyl-2-O-[4’-O-(α-D-glucopyranosyl)-β-Dglucopyranosyl]- sn-glycerol (DMMA). (B). Chemical structure of a lipopolysaccharide LPS from deep rough mutant Salmonella minnesota R595 (LPS Re).

The driving force for the formation of a mesophase of amphiphile molecules is a micro-phase separation. This leads to an aggregate structure (Fig. **2** and Fig. **3**) with separated regions for the lipophilic (hydrophobe) and hydrophilic (polar) moieties.

## 2 SELF-ASSEMBLY AND AGGREGATION PROPERTIES OF GLYCOLIPIDS

Similar to other amphiphilic molecules such as phospholipids, glycolipids tend to aggregate in aqueous dispersions due to the minimization of the Gibbs free energy [10, 11]. The concentration at which such aggregation takes place is called the critical micellar concentration (CMC), below this value the molecules are present as monomers [12]. A different terminus, critical aggregate concentration (CAC), is the concentration at which monomers spontaneously may form aggregates which, however, decompose again. Thus, the CAC always lies below the CMC. 

Above the CMC, with increasing glycolipid concen-tration, the monomer concentration remains constant, or is even reduced in the case of negatively charged amphiphiles, and additional molecules are incorporated into the aggregated form [13]. Thus, above the CMC aggregate as well as monomers are present and in a state of equilibrium. An important characteristic is the type of aggregate structure formed above the CMC. It depends on the relative sizes and geometries of the hydrophilic and hydrophobic moiety; this was considered by Israelachvili who introduced a dimen-sionless shape or packing parameter S = v / ( a_0_^.^l_c_) = a_h_ / a_0_ (v = molecular volume of the hydrophobic moiety, l_c_ length of the fully extended hydrophobic moiety, a_0_, a_h_ cross-sectional areas of the hydrophilic and hydrophobic moiety, respectively). This parameter is also known as CPP (critical packaging parameter) or IPF (Israelachvili packaging factor) [13].

If the value of S is known, the resulting aggregate structures can readily be deduced. For S < ½ micellar structures (not to mix with the term micelle as synonym for aggregate) are adopted, in particular cases the direct H_I_ phase is favoured. In each case, the acyl chain moiety is directed inwards, and the hydrophilic part outwards. Between S = ½ and 1, lamellar structures in the form of unilamellar or multilamellar structures are favoured. Whether a particular glycolipid adopts an uni- or multilamellar structure is a complex problem, which depends, among others, on geometrical constrains, on the charge distribution in the head group, the kind of counter ions, and the hydration properties of the glycolipid. 

Above S = 1 inverted structures such as inverted hexagonal H_II_ or cubic structures of type II (so far not described by Israelachvili (1991) [13]) are formed; the acyl chains are directed outwards, and the hydrophilic moiety inwards, see Luzzati *et al.,* [14, 15]. A critical region is the range around S = 1, where various phases may coexist and in which small extrinsic changes such as hydration, ion concentration or temperature change may lead to a phase transition. Since the packing parameters cannot be estimated with sufficient precision, physical techniques such as small-angle scattering with X-rays (SAXS) or neutrons (SANS) must be applied for a reliable determination of the structures [16]. An overview of the different possible aggregate structures is given in Fig. (**2**)**.**

A second parameter of aggregated structures is of importance, namely the state of order or fluidity of the acyl chains. Basically, two states, the gel (β) and the liquid-crystalline (α) phase can be adopted, and between these a (pseudo) first-order transition can be observed. In the gel phase the acyl chains are ordered, being in the all-trans configuration, while the liquid-crystalline (fluid) phase is much less ordered (higher fluidity) due to the introduction of more and more *gauche-*conformers. This phase transition at a glycolipid-specific temperature T_m_ can take place within one and the same aggregate structure, but can also be connected with a change of the aggregate structure. It is known that not all aggregate structures can occur within both phases. 

The H_II_ structure has only been observed in the liquid-crystalline state. In the gel phase the acyl chains are not flexible enough to adapt to the high curvature of the H_II_ structure [17-20]. 

It was assumed earlier that also cubic structures are restricted to the fluid phase [14]. This, however, could not be confirmed in the case of glycolipids from bacterial origin [21, 22]. 

The value of T_m_ is governed by various parameters: Length and the degree of saturation of the hydrocarbon chains moiety, the kind of interface region, and type of head group, as well as hydration properties and solution properties (e.g. pH, ionic strength, presence of divalent cations) [23]. Another important parameter is the degree of water binding. For glycolipids with head groups/interface regions of low hydrating capacity T_m _is higher than for those with a well hydrated backbone. Therefore, the lyotropic behaviour is an essential parameter for a physico-chemical characterisation of glycolipids. 

For an understanding of the physico-chemistry of the self-organisation of glycolipids the establishment of complete phase diagrams is necessary [24]. This means that the lyotropism – dependence on water content – and the thermotropism – dependence on temperature – has to be evaluated (Tables **1A** and **1B**). For charged lipids, which are frequently found in nature, also the dependence on the type of counter-ions such as Na^+^, Mg^2+^, and Ca^2+^ is an important parameter [25, 26]. Such structural polymorphism–complete phase diagrams with temperature, water content, and cation concentration as parameters, has been published for the glycolipid lipid A and LPS Re from *Salmonella minnesota* [21, 22]. It was shown that these compounds have a clear dependence on water content in the aggregate structures as well as on the phase transition temperature T_m _(see text and figures below). 

As examples for the compositions of glycolipids, the chemical structures of dimyristoyl-maltoside DMMA (Fig. **3A**) and a lipopolysaccharide from deep rough mutant R595 (LPS Re, Fig. **3B**) are presented.

For the measurements of the CMC fluorescence spectroscopy or light scattering techniques have been applied in many cases [27]. The former is based on the fact that suitable dyes such as *N*-phenyl-naphtylamine (NPN) or anilino-naphtalene sulfonate (ANS) have a specific fluorescence at a fixed wavelength when incorporated into aggregates which shift or disappear upon disaggregation [28]. Using light scattering techniques, the concentration of the amphipiles are increased step wise and the formation of amphiphile aggregates at the CMC is observed by a strong increase in scattering intensity. A different method is the CMC determination by a monolayer technique. The successive addition of the amphiphilic molecules to the subphase leads to an increase of the monolayer pressure until attaining the CMC [10, 27].

The described methods are limited to CMC down to the micro-molar concentration range; therefore, for samples with CMC lying at lower concentrations such as in the case of many diacylated glycolipids, these techniques do not apply. For these, special approaches such as the use of radioactively labelled compounds have to be used. In the case of glyco-lipids with a sufficient number and length of acyl chains, similar as in the case of phospholipids, data on the CMC are very scarce, because only very limited and sophisticated methods may be successful. For example, for the glycolipid lipid IVa (a precursor of lipid A from enterobacterial strains with four C-14 acyl chains) a CMC of < 10^-7^ M, obtained with Laser light scattering [29], was indicated. 

Another study on rough and smooth LPS and hexaacyl lipid A, obtained with fluorescent dyes and light scattering, indicates a CMC in the micro-molar range (≈ µM) for all these samples, irrespective of the length of the sugar chain [30]. This value, however, corresponds to the sensitivity of the method and does not reflect the CMC, which, of course, must differ for the different compounds by orders of magnitude due to the variations in the sugar chains (having the same lipid moiety). Sasaki and White (2008) [31] have applied high energy dynamic laser light scattering of highly purified LPS Re and have found a drastic decrease of the ‘hydrodynamic radius’ of the aggregates in the range of some nanomolar. The authors, however, did not present the resolution limit of their technique, which might indicate that the true CMC is even much lower. Summarizing all information on the CMC of LPS, it can be assumed that the CMC for lipid A and LPS Re is in the range significantly below 10^-9 ^M similar as argued by Brandenburg and Wiese (2004) [32]. 

For the determination of glycolipid aggregate structures small-angle X-ray (SAXS) or neutron scattering (SANS) are the methods of choice. With the current brilliance of synchrotron radiation the SAXS experiment can be readily performed whereas neutron radiation with a sufficiently high intensity is less available. The diffraction patterns obtained in this way can be analysed with respect to the positions of the single peaks being characteristic for the single phases [27]. With regard to lyotropism, it could be shown for lipid A and various rough mutant LPS from *S. minnesota*, that these compounds show only a multilamellar structure at low (0 to 40 %) water content, which passes into a unilamellar or cubic structure at high water content ( > 60 %) [21, 22, 33]. Similarly, the aggregate structure changes upon heating, being unilamellar, mixed unilamellar/cubic in the gel phase of lipid A, convert into a hexagonal H_II_ phase at temperatures significantly above T_m _(45 °C).

The biological importance of the phase states and the transitions between them at a particular temperature depends of course on the aggregates structures within the single phases. Thus, for example, the L ↔ Q transition is kinetically inhibited, and grows only slowly, while the L ↔ H transition takes place directly and rapidly. In this context also the reversibility must be seen, which takes place most readily for the latter, but in a delayed mode for the former transition. Various biological processes are dependent on the phase states and temperatures of the transitions, for example endo- and exocytotic effects. Also, processes such as cell-cell interaction, cell adhesion, and binding and recognition processes by receptors are governed by the state of order of the membrane matrix and its constituents (see chapter VI).

## 3 PHYSICAL TECHNIQUES FOR THE DETERMI-NATION OF GLYCOLIPID ORGANISATION

In the following chapters, some common and most powerful physico-chemical methods for the determination and characterization of lipid organization are presented. Also methods for the analysis of phases and phase transitions are shown (for more details see for example Cevc 1993 [34], Shechter 2004 [35], Winter and Noll 1998 [27]).

### 3.1 Differential Scanning Calorimetry (DSC)

Using the DSC technique, phase transitions can be monitored which are related to enthalpy contributions. A fully hydrated phospholipid, like DPPC (Di-Palmitoyl-*sn*-glycero-phosphatidyl-choline) shows the thermotropic induction of three phases separated by two peaks: (i) endothermic changes in enthalpy of the transition from L_β’_-phase to the P_β’_-phase at ca 36 °C, and (ii) the phase transition to the liquid-crystalline L_α_-phase at 41.5 °C (data not shown) [23]. 

The phase transitions observed by DSC are of first or pseudo-first order [36]. Thus, in the heating scan the melting of hydrocarbon chains from a gel to a liquid-crystalline phase is related to an endothermic phase transition (endotherm peak). A subsequent cooling shows the appearance of an exothermic peak [37-39]. The re-crystallisation of the hydrocarbon chain can be quite reversible, as shown in Fig. (**4A**) or can be connected to a strong hysteresis [40, 41]. The reason for the non-reversibility of the phase transitions has been reviewed by Tenchov [39] and the different mechanisms are described extensively (for more details see Tenchov 1991 [39]). 

In cases, in which the phase transition is not due to changes in enthalpy contributions, no “peak” is observed in the DSC thermogram. For example, the lamellar to inverted hexagonal phase transition (H_II_) phase is accompanied by a very small endotherm change in enthalpy, usually in the range of 10 % of the main gel-to-liquid-crystalline phase transition [39] and therefore it is not always detected [40].

A reason for the relatively small phase transition enthalpies is related to the fact that lamellar to non-lamellar phase transitions usually take place within the liquid-crystalline phase [42, 43]. Curvature as well as a change of effective packaging of the lipid molecules has also to be considered for understanding the thermodynamics of the L_α_- H_II_ phase transition.

This has extensively been investigated by calorimetry for phosphatidylethanolamine lipids [44-46]. Although, being very small, the L_α_-H_II_ phase transition enthalpy is expected to be of first order due to symmetry considerations, and therefore it is detected [47]. 

The phase transition from the liquid-crystalline to a cubic phase are hardly observed by DSC, however can be easily detected using SAXS [48]. Monoolein in water, for example shows a small endotherm peak in the heating DSC scan which is related to a lamellar to cubic phase transition [49].

Lamellar to non-lamellar phase transitions have also been observed upon heating glycolipids, for examples see Table **1** [39]. In most cases these phase transitions are observed by SAXS (see below).

Current high sensitive differential calorimeters are of adiabatic type and allow the measurement of heat changes in the micro-calorimetry range DSC is certainly one of the most sensitive methods for the investigation of phase transitions of lipids [23, 36, 50]. 

Fig. (**4B**) shows the thermograms of a fully hydrated glycolipid (Fig. **3A**) stored at 4 °C. As a function of storage time at 4 °C, new phases are formed. These phases are observed at higher temperatures. The reason for the formation of these new phases is highly related to the dehydration of the disaccharide head group of the glycolipid as it has been observed for other lipids [25, 40, 51]. The authors investigated cis-unsaturated galactosylceramides with varying incubation times, which caused differently stable packing motivs and thus nucleation events that promote transbilayer interdigitation. However, from the slow kinetics of the process, the authors conclude that these monounsaturated chains common in galactoceramides may inhibit transbilayer interdigitation under physiological conditions. 

From the DSC results a number of thermodynamic parameters are obtained, e.g. onset temperature of the phase transition, phase transition temperature, phase transition enthalpy Based on these data and using thermodynamic rules, additional parameters like the phase transition entropy (∆S_cal_ = ∆H_cal_/T_m_) can be calculated.

The cooperativity of the phase transition can also be estimated from the heat capacity profile. This is done by the calculation of the van’t Hoff enthalpy (∆H_vH_), which is a measure of the sharpness, i.e., cooperativity of the phase transition. In the case, the width (∆T_1/2_) of the phase transition is quite small in relation to the maximal temperature of the heat capacity (T_m_), the van’t Hoff enthalpy can be obtained by the following approximation (Blume 1988 [36] and references cited therein):

∆H_vH_ ≈ 6.9^.^T_m_^2^ /∆T_1/2_

For the example shown in Fig. (**4A**) (heating scan of DMMA) ÄH_vH_ = 612 kJ/mol. Thus, ÄH_vH_ >>> ÄH_cal_ (the calorimetric enthalpy). Based on these two parameters the so-called cooperative unit c.u. is calculated as: c.u. = ∆H_vH_ / ∆H_cal_. The cooperative unit is an estimate for the number of lipid molecules from which the phase change is nucleated [27]. Or in other words, this is the number of molecules which change together from one state to another, e.g. from a gel phase to a liquid-crystalline phase. For the glycolipid phase transition shown in Fig. (**4A**), the c.u. is 19. The cooperative unit can also be seen as a measure of the degree of intermolecular cooperation between the lipid molecules. For phospholipids like DPPC (dipalmitoyl-phosphatidyl-choline) the cooperative unit is much higher and reaches values of approximately 600 [50]. In the case a phase transition occurs completely cooperative, the 1^st^ order phase transition of an absolutely pure compound has an infinite c.u. [36, 52]. In the case of a completely non-cooperative equilibrium process the c.u. of the phase transition is unity.

Information on lipid-water interactions are also available from DSC by determining the apparent molar heat capacities ^ϕ^Cp [36, 51]. In general, hydrations of polar lipid head groups give negative contributions to the total heat capacity. This effect occurs due to the orientation and immobilisation of water molecules in the close vicinity of the polar head group. On the other hand, hydrophobic hydration of non-polar molecule groups like methylene units of the hydrocarbon chains are connected to a positive contribution to the total heat capacity, which is related to entropic effects [36, 51].

### 3.2 Infrared Spectroscopy (IR)

The gel to liquid-crystalline phase transition can be evaluated in several ways, by the application of Fourier-transform infrared spectroscopy (FTIR), differential scanning calorimetry (DSC, see above), and fluorescence polarisation [27, 28, 35]. FTIR is one of the most familiar techniques in glycolipid science (see, for example Brandenburg and Seydel, 1998 [53], Brandenburg and Seydel 2002 [54]. It allows the determination of intra- and intermolecular conformations by studying the motional freedom of infrared radiation absorbing groups within the molecules and changes due to perturbations like temperature, pH, solutes, hydrogen, and salt binding. Variations of these parameters not only act intramolecularly, but may lead to different intermolecular interactions with possible conformational changes of the secondary structure – identical molecules in one plane and/or the tertiary structure of the three-dimensional aggregates. Therefore, FTIR not necessarily allows the differentiation between pure binding of salt or solute molecules and the accompanying conformational change. In this case, complementary methods such as X-ray scattering (see below) have to be applied.

Using FTIR particular vibrational bands from the hydrophobic moiety sensitive to the state of order of the acyl chains can be taken [55]. In this way it is possible to investigate different part of the molecules due to the vibrational characteristics of the molecular groups. For phase transition measurements, the lipid methylene stretching vibrations v_as _(as: antisymmetric) and v_s_ (CH_2_) vs: symmetric) are the most suitable bands. In particular, the latter band is taken in most cases to monitor phase changes since it is less disturbed as the antisymmetric mode. 

The peak position of the symmetric stretching vibration of the methylene groups v_s_(CH_2_) lies around 2847 to 2848 cm^-1^ for crystalline phases such as in ceramides, around 2850 cm^-1^ for the “normal” gel phase, and at 2853 to 2854 cm^-1^ in the liquid-crystalline phase [53, 56, 57].

Information on the organisation of the hydrocarbon chains can be derived from the analysis of the methylene scissoring or rocking band vibrations [57, 58]. The shape of these bands as well as the position is indicative for whether the hydrocarbon chains are aligned in a hexagonal, triclinic or orthorhombic lattice [55, 58, 59].

All-trans hydrocarbon chains aligned parallel and packed in a triclinic subcell lattice reveal a methylene rocking band maximum at ca 718 cm^-1^, whereas a band maximum at 721 cm^-1^ is related to a hexagonal methylene all-trans chain packing and/or orientational disorder of the hydrocarbon chains [38]. This can be discriminated by the additional analysis of the symmetric or antisymmetric methylene stretching vibrations. In the case a splitting of the band into two components located at 719 and 731 cm^-1^ is observed, an organisation of the hydrocarbon chain into an orthorhombic subcell lattice with the planes of ordered all-trans acyl chains being arranged perpendicular to each other is likely [55].

The wavenumber characteristic for the scissoring vibration of a hexagonal packing is located at ca 1468 cm^-1^, while triclinic packing is marked by a shift to 1473 cm^-1^. A splitting of the scissoring mode in two components located at 1462 cm^-1^ and 1472 cm^-1^ is typical for acyl chains arranged in an orthorhombic lattice [58]. 

Hydrated DMMA (Fig. **3A**), stored for a few days at 4°C, shows a phase formation with the acyl chains organized very tightly in a triclin-like lattice [40, 41, 60, 61]. 

Information with regards to the organisation and hydration of the polar sugar head group can also be derived from infrared spectra by analysing corresponding vibrations like C-O absorption etc. [58].

As stated above, glycolipids exhibit a pronounced lyotropism. It was found that T_m_ decreases with increasing water content, lying at more than 50 °C at low and around 43 °C at high water content for hexaacyl lipid A from *S. minnesota* [21, 62]. This is also valid for different glycolipids such as dimyristoyl melibioside with a T_m _of 24 °C at 98 % and 30 °C at 62 % water content [40]. 

What might be clear from the chapter above, additional transitions may take place, for example from a lamellar into an inverted phase such as cubic and H_II_, which is connected with much less enthalpy change, only 5 to 10 % of the main phase transition. 

For example, Fig. (**5A**) shows ‘normal’ gel to liquid-crystalline phase transition behaviour of various salt forms of LPS Re and lipid A. As can be seen, the T_m_ values, indicated in Fig. (**5A**), depend strongly on the sample (lipid A is *a priori* higher than LPS Re) and on the salt forms. In particular, the heavy salts Ca^2+^ und Ba^2+^ shift the T_m_ values to higher temperatures, and does not even pass into the liquid-crystalline phase with the Ca^2+^ salt form of both samples, for which typical wavenumber values of 2852 to 2853 cm^-1^ are observed [53]. It should be noted that for these heavy salt forms the LPS lose their ability to induce cytokines in immunocompetent cells [59], making these observations relevant for an understanding of biological effects. Further examples are given in Fig. (**5B**) for dimyristoylmaltoside and Fig. (**5C**) for the monoacylated (stearyl) glucosides and galactosides in α- and β-linkage. The compounds exhibit a metastable behaviour: After cooling for a certain time at ca 4 °C the phase transition has a value around 41°C, without longer cooling this value shifts down to 34 °C (see the corresponding DSC data in Fig. (**4B**). Concomitant with this process, the wavenumbers below T_m_ are quite different, around 2850 or 2849 cm^-1^, respectively. Here, it becomes clear that also the aggregate structures must be known for an unequivocal assignment. The later presented SAXS data will show, that the transition at the higher temperature is from a crystalline into the fluid, liquid-crystalline phase, whereas that at the lower T_m_ corresponds to the ‘normal’ gel to liquid-crystalline phase transition [40]. In Fig. (**5C**) in contrast, the transition can readily be interpreted (see later for SAXS data) to pass from an interdigitated phase into the ‘normal’ L_α_ phase. These data demonstrated that the wavenumber values of the band positions of v_s_ (CH_2_) do not necessarily allow direct statements about the underlying aggregate structure [62].

In previous reports Mannock *et al.* [63-65] as well as Hinz *et al.,* [66] have performed systematic studies of glycolipid membrane systems. By analysis of ether-linked alkyl chains with 10 to 18 carbon atoms and mono- to trisaccharide head groups the phase transition behaviour could systematically be determined [66]. In accordance with the findings the results showed that galactolipids strongly tend to convert directly from the well-ordered crystalline L_c_ into the inverted hexagonal phase (see also Mannock and McElhaney, 1991 [63]). 

Von Minden *et al.,* (2000) [67] investigated the thermotropic phase behaviour of long chain monoalkyl glycopyranosides with different disaccharide head groups and found a distinct dependence of T_m_ on the kind of sugar head group as well as on the type of linkage (α or β). This is shown in Fig. (**5D**) for C18 carbon chains and the head groups maltose (Mal), melibiose (Mel), cellobiose (Cel), and lactose (Lac). In the α-linkage, the Cel melts at highest T_m_, followed by the Lac and the Mel compounds. In the β-linkage, the comparison of the Lac- with the Mal- containing compound shows in this case a much higher T_m _for the former compound. These data are indicative of the packing constraints of the head groups. The Cel-containing backbone essentially has a very flat geometrical extension, while the Lac-containing sample is less flat due to the galactose as second monosaccharide (OH group at position 4 showing upwards). The Mel-containing sample with the 1(6 linkage of the Glu-Gal, in contrast, has a much higher space requirement with a conical shape thus leading to a much less dense packing of the glycolipid. Also, it was reported that the supramolecular aggregate structures of these compounds differ strongly [67].

### 3.3 Fluorescence Spectroscopy 

Fluorescence spectroscopy is a highly sensitive technique for the investigation of self-organised amphiphiles like glycolipids as well as for studying biological membranes [58]. Various properties of assembled lipids can be studied using fluorescence spectroscopy and related applications [68]. For instance, using polarised fluorescence [28] the orientation and rotational mobility of lipids or the micro-viscosity of internal region of membranes can be determined. The combination of fluorescence intensity and polarized fluorescence allows the analysis of phase transitions and lateral phase separations, as well as the organization of lipids in the vicinity of e.g. proteins [68].

In order to be able to use fluorescence spectroscopy for the investigation of lipids, external fluorophores such as diphenylhexatriene (DPH) are used. There are a number of fluorescent probes available with different physico-chemical properties, suitable for a large number of fluorescent experiments [28]. The choice of the fluorophore depends on the lipid organisation information of interest. 

The diverse probes available are designed to 1) label a specific region of an organised lipid assembly and 2) should be selected to have emission properties which are particularly sensitive with respect to the lipid domains that are analysed [28, 58, 68]. Thus, the hydrophobicity of the probes, besides their fluorescent properties, is an important criterion of selection. Nonpolar fluorescent probes that can be used are for example perylene, pyrene or DPH. Various polar probes are available like: 1-Anilino-8-naphtalene sulfonate (ANS) or N-phenyl-1-naphtylamine (NPN) [28, 68].

The theory of lipid bilayer phase transition as detected by using fluorophores has been studied for years [28, 69, 70]. For more details on the technique please consult for example Berlin and Sainz 1984 [70], or textbooks like Valeur 2002 [68], Hof, Hutterer, Fidler 2005 [71], Lakowicz 2006 [28].

Since extrinsic fluorescence spectroscopy is not a label-free technique, it is often argued that the data obtained in this way do not always reflect and give statements about the absolute state of lipid order, and therefore comparing the results obtained by various methods should be critically considered and evaluated.

### 3.4 Monolayer Studies 

The behaviour of amphiphiles at the air-water-interface can be determined by the film-balance technique developed by Pockles (1891) [72] and Langmuir (1917) [73].

Amphiphilic molecules like glycolipids form a mono-layer when distributed between the aqueous bulk phase and the air-water-interface in solution. This distribution depends on the physico-chemical properties of the lipid such as its hydrophobicity. When distributed at the air-water-interface, amphiphilic compounds arrange in a monomolecular layer with their polar head group(s) facing towards the water phase.

The space required by the head group can be measured with a film balance [26, 74]. The surface pressure **π** caused by a compressed monolayer is measured by a force meter [74]. This surface pressure **π **is described as the deviation between the subphase γ_0_ covered with a monolayer and the uncovered subphase γ:

**π** = ∆γ = γ_0_ – γ

The surface pressure **π** (in mN/m) is related to the surface area A (Å^2^/molecule), which is occupied by a single molecule. Data about the phase behaviour, the orientation on surfaces or interfaces or interactions of the amphiphiles among each other can be derived by the form of the measured isotherm [74].

Fig. (**6**) represents a surface pressure/area isotherm of the glycolipid DMMA (Fig. **3A**) measured at two different sub-phases at 20 °C: water and 20 mM HEPES (buffer). Fig. (**6**) shows that the liquid expanded state and the formation of a plateau is very well expressed. This happens when equilibrium is formed between a liquid expanded and a liquid condensed state. This lipid forms a film with the lipid molecules extremely tightly packed [40].

### 3.5 Polarisation Microscopy 

Polarising microscopy is a well established method to investigate the properties of anisotropic compounds [75, 76]. Being widely used in mineralogy and petrology it has become a standard method to investigate the phase behaviour of liquid-crystals. 

According to Snellius’s law of refraction, optical anisotropic compounds show birefringence if placed and observed between crossed polarisers. This means that a beam of light that passes through an anisotropic material in a direction different to the material’s optical axis is refracted into two different directions [34, 75]. These two beams show an optical retardation which leads to a specific texture that can be seen trough a polarising microscope. These textures are specific for certain thermotropic and lyotropic liquid-crystalline phases [75, 77-79]. 

Additionally to the setup of an ordinary microscope, microscope systems are available that allow to place the sample in polarizing microscope equipped with a hot stage (heating device) between two polarisers of which one can be altered in direction [27]. 

To investigate the thermotropic phase behaviour of a liquid-crystal, a small sample of the compound on a glass slide is placed into the heating measuring sample chamber. While the temperature is raised or lowered at a specific scan rate, alterations of the sample texture can be observed through the polarisation microscope.

#### 3.5.1 Thermotropic Phases Observed by Polarisation Microscopy

Glycolipids are known to form a range of thermotropic smectic, columnar and cubic phases. Examples of the textures are shown in the following figures (Figs. (**7**-**11**)).

Figs. (**7**-**11**) show specific textures of a smectic A phase. Smectic phases are layered structures [5]. The molecules form layers in which every molecule can move within its own layer, but cannot change its general direction (up or down) nor can the molecules leave the layer. These phases form a variety of characteristic textures. Fig. (**7A-1**) shows a smectic Grandjean texture (“Stufentropfen”). In Fig. (**7B-1**) a fan-shape texture is shown, whereas. Fig. (**7C-1**) shows a pearl-chain texture which often is observed at higher temperatures and maltese cross texture which often is observed on cooling the sample.

In columnar phases the molecules are ordered in stacks or columns. These aggregates form characteristic textures like the textures shown in Fig. (**8A-1**), Fig. (**8B-1**) and Fig. (**8C-1**).

Cubic phases are not easily observed using polarisation microscopy, due to their isotropic texture. In a cubic phase all molecules are ordered in body-centered or face-centered cubic lattices. That means that the light passing trough the sample is refracted in every possible direction, so the sample appears isotropic. A cubic phase differs from the isotropic melt only by its low viscosity.

Fig. (**9A-1**) shows an example of a pure cubic phase which is still visible by a special surface structure. A thin film has only partially contact to the cover slide. The upper left area is fully covered by the slide and looks like a liquid whereas the main part is not covered and the surface of the film shows geometrical forms at the interface to the air.

Fig. (**9B-1**) shows a transition from a hexagonal phase to a cubic phase. In this case the cubic phase forms rectangular isotropic textures. Cubic phases are best observed during the transition from a non isotropic phase, because the cubic phases themselves usually show no textures.

#### 3.5.2 Lyotropic Phases Observed by Polarisation Microscopy 

The method of choice to investigate the lyotropic phase behaviour with a polarizing microscope is the contact preparation method [80]. Therefore, the sample is placed on a glass slide and is covered with a coverslip. Then the sample is heated up to its melting point and cooled down again in order to generate a coherent surface. After cooling to room temperature a solvent (e.g. water) is placed on the edge of the glass slide. The solvent then is drawn between the slide and the coverslip by capillary force and thus gets in contact with the sample. At the contact area between solvent and sample, the solvent diffuses into the sample, so a certain grade of concentration is achieved. Along this concentration grade the lyotropic phases of the sample can be observed. If necessary, the sample can be moderately heated to detect kinetically hindered phases like the cubic phases. A better way to detect lyotropic cubic phases is to expose the sample to a water saturated atmosphere (e.g. in an excicator) for a certain time to let water diffuse into the sample.

Fig. (**10A-1**) shows the lyotropic phase sequence of the glycolipid MalC_12_N_3_ (Fig. (**10A-1**)). The phase sequence starts with water (**A**), then a broad hexagonal H_I_ phase can be observed (**B**) followed by a bicontinuous cubic phase V_I_ (**C**) and the pure compound in a thermotropic smectic phase (**D**). 

The phase sequence of GenC_12_N_3_ is shown in Fig. (**10B-1**). The sequence starts in the upper left corner with the pure compound in a glass phase (**A**) followed by a hexagonal H_I_ phase (**B**) and water (**C**).

In Fig. (**10C-1**) the phase behaviour of LacCerPiv, a sphingosine analogous glycolipid, is shown. Beginning in the lower left corner with water, this compound shows a hexagonal (H_I_) phase followed by a bicontinuous cubic phase (V_1_) and a lamellar phase (L_α_) and a SmA phase the pure compound.

Sometimes a second cubic phase can be seen. In Fig. (**11A-1**) the phase sequence of Mal-OC_3_-C_12_ shown, beginning with water on the left, followed by a discontinuous cubic phase (I_1_), a broad hexagonal phase (H_II_), a bicontinuous cubic phase (V_1_) and the pure compound with a smectic A (SmA).

**Fig. (12):**
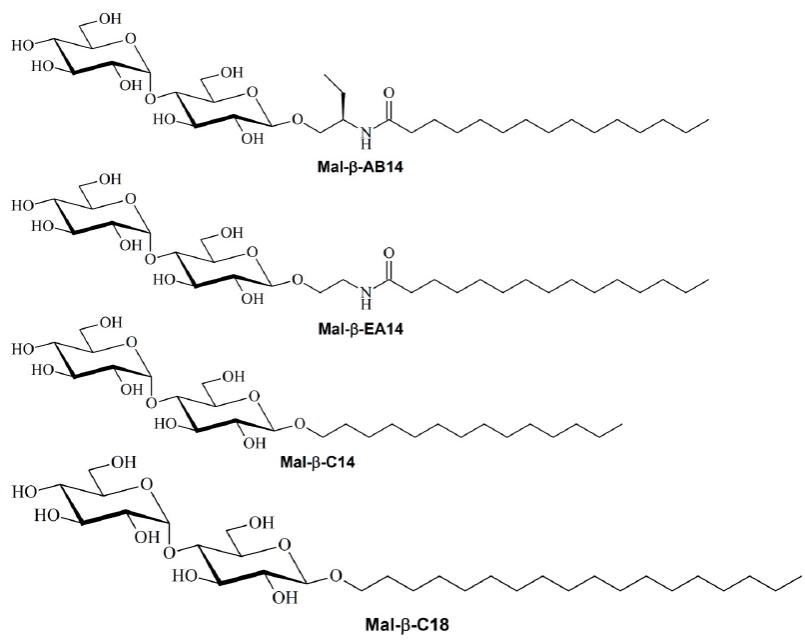
Molecular structures of the branched Mal-β-AB14, unbranched Mal-β-EA14 and the normal n-alkyl maltosides Mal-β-C14 and Mal-β-C18, showing the main structural differences.

The thermotropic and lyoptropic phase transitions of selected glycolipids (Fig. **12**) are summarised in Table **1**. For a clear phase assignment additional physico-chemical methods are used (see next section).

## 4 PHYSICAL TECHNIQUES FOR THE DETERMINATION OF AGGREGATE STRUCTURES

The method of choice to determine aggregate structures of glycolipids is the application of X-ray or neutron scattering (= X-ray or neutron diffraction) [27]. Here it is focussed mainly on small-angle X-ray scattering (SAXS) due to the availability of high-brilliance synchrotron sources. Usually a differentiation between the small-angle region and the wide-angle region (SAXS= small-angle X-ray scattering, WAXS = wide-angle X-ray scattering) is made [27].

Applying this technique, the three-dimensional organization of biomolecules can be calculated according to the Laue or the Bragg equation [27]. X-ray scattering patterns are recorded in the case of SAXS in the range 0.01 < s < 1 nm^-1^, and in the case of WAXS (wide-angle X-ray scattering) in the range 2 < s < 5 nm^-1^ (scattering vector s = 1/d, d = spacings of the sample, s = 2 sin θ/λ, 2θ scattering angle and λ the wavelength, usually at 0.15 nm). For the X-ray diffraction patterns shown here, the measurements were performed at the European Molecular Biology Laboratory (EMBL) outstation at the Hamburg synchrotron radiation facility HASYLAB using the double-focussing mono-chromator-mirror camera X33 [81]. The diffraction patterns were recorded at a specific temperature with exposure times of 1 min using a linear detector with delay line readout (‘Gabriel’-detector) or, as new instrumentation - an image plate detector with online readout (MAR345, MarResearch, Norderstedt/Germany) [82]. The s-axis was calibrated with Ag-behenate which has a periodicity of 58.4 nm. The diffraction patterns were evaluated as described previously [22, 83, 84], assigning the spacing ratios of the main scattering maxima to defined three-dimensional structures.

The scattered X-ray intensity (I = |F|^2^) can be written as product of the structure factor and the form factor. In the case of lamellar structures, this expression can be written explicitly:

F=∑0N−1e2πRnd∫0dρ(x)e2πiRxdx

The integral represents the charge distribution along one bilayer with the periodicity d, and the first term is the summation over N stacks. 

The SAXS range allows the determination of the long-range order, i.e., the periodicities of the aggregate structures: The most relevant of these structures are direct micellar, lamellar, cubic and hexagonal (direct H_I _and inverted H_II_), and are presented in Fig. (**2**). They are characterized by the following features (see also chapter ‘Self-assembly and aggregate properties of glycolipids’):

(1) Micellar structures with acyl chains inside and head groups outside. The simplest forms would be spherical micelles and more complex forms are the H_I_ structures (see Vill *et al.,* 1989 [85], Brandenburg *et al.,* 2000 [62] and 1998 [86]). 

An example for SAXS patterns of spherical micelles which have so far not been described in literature are given in Fig. (**13**) (monoacylated lactoside Lac-β-OC_2_-OC_14_), and of the direct H_I_ structure in Fig. (**14**) (triacylated lipid A, derived from *E. coli* lipopoly-saccharide). The latter spectrum consists of the reflexions at 5.10, 4.42, and 3.31 nm which are related to the periodicity at 8.78 nm as the √3-, √4-, and √7-fold. The former diffraction pattern can be described as the Fourier-transform of a sphere, i.e., in the above equation only the form factor gives a contribution (N=1, this means the structure factor equals zero). 

(2) Lamellar structures show a three-dimensional organisation with 1-dimensional symmetry.

*A) Unilamellar:* Here, usually only one broad diffraction band is observed. 

In Fig. (**15A**) an example of a glycolipid, in which only the lipid bilayer, an unilamellar structure in an undefined geometry, is expressed, the SAXS pattern of a lipopolysaccharide from *S. minnesota *Ra-mutant (strain R60) is shown.

*B) Multilamellar:* The reflections are grouped at equidistant ratios, i.e., 1, 1/2, 1/3, 1/4, etc. of the lamellar repeat distance dB_l _(see equation above). As an example for a multilamellar stack the diffraction pattern of a lipid A preparation in the presence of an antimicrobial peptide (EU#36 based on human lactoferrin) is presented in Figs. (**15B** and **16**). 

Furthermore, also the glycolipid monoacyl maltoside (Mal-β-NH-COC_15_), as ester-bound, is organized into multilamellae. 

(3) Cubic: These phases have a three-dimensional symmetry. The different space groups of these non-lamellar three-dimensional structures differ in the ratio of their spacings. The relation between the reciprocal spacing sB_hklB_ = 1/dB_hklB_ and lattice constant a is

sB_hklB_ = [(hP^2P^ + kP^2P^+ lP^2P^) / a ]^1/2^

(hkl = Miller indices of the corresponding set of plane).

According to the geometry, cubic phases may be of type I (the cross-section of the hydrophilic moiety is larger than that of the hydrophobic moiety) or of type II (the reversed situation of type I). 

For lipid samples, including glycolipids, the bicontinuous cubic phases Q^224^ (Pn3m), Q^229^ (Im3m), and Q^230^ (Ia3d) are of particular importance [14]. It has been shown that lipid A under near physiological conditions has a preference for cubic phases, in particular Q^224^ and Q^229^ [86]. A very impressive example of a cubic phase for a glycolipid is given in Fig. (**17**) for the diacylated disaccharide maltose-1,3-oleyl (see Milkereit *et al.,* 2005 [87-89]), for which the occurrence of reflections at 1/√2, 1/√3, 1/√4, 1/√6, 1/√8 , 1/√9 ,1/√10 , 1/√12 , 1/√14, and 1/√16 of a periodicity at 11.65 nm are characteristic for the cubic phase Q^224^.

(4) Hexagonal: This is a structure with 2-dimensional symmetry. The relation between s and the basic vector a is:

shk=2√h^2^+k^2^–hk/ a √3 

(s_10 _=2/a √3=1/d_h_, 

d_h _= Spacing of the basic periodicity).

Hexagonal structures may occur with the lipid moiety inside and the polar head group outside (H_I_) or vice versa (H_II_). Inverted hexagonal phases are frequently found for glycolipids in the liquid-crystalline phase at high temperatures. Here, two examples are given in different presentations. 

In Fig. (**18A**), a SAXS pattern measured at 80 °C is shown for a highly purified lipid A preparation, isolated from LPS from E coli, exhibiting reflections that occur at the 1/√3, 1/√4, 1/√7, 1/√9, 1/√12, and 1/√13 of the periodicity at 5.88 nm. The data for the compound dioleoyl-galactoside is shown in Fig. (**18B**) in which the electron density distribution of the H_II_ phase at 40 and 60 °C is presented. 

Beside these basic structures, also particular phases are observed. To these belong interdigitated phases for monoacylated saccharides. Such an interdigitated phase is presented in Fig. (**19**) for stearoyl-α-galactose in the temperature range 20-60 °C, which converts into a ‘normal’ L_α_ phase at 80 °C [80].

**Fig. (20):**
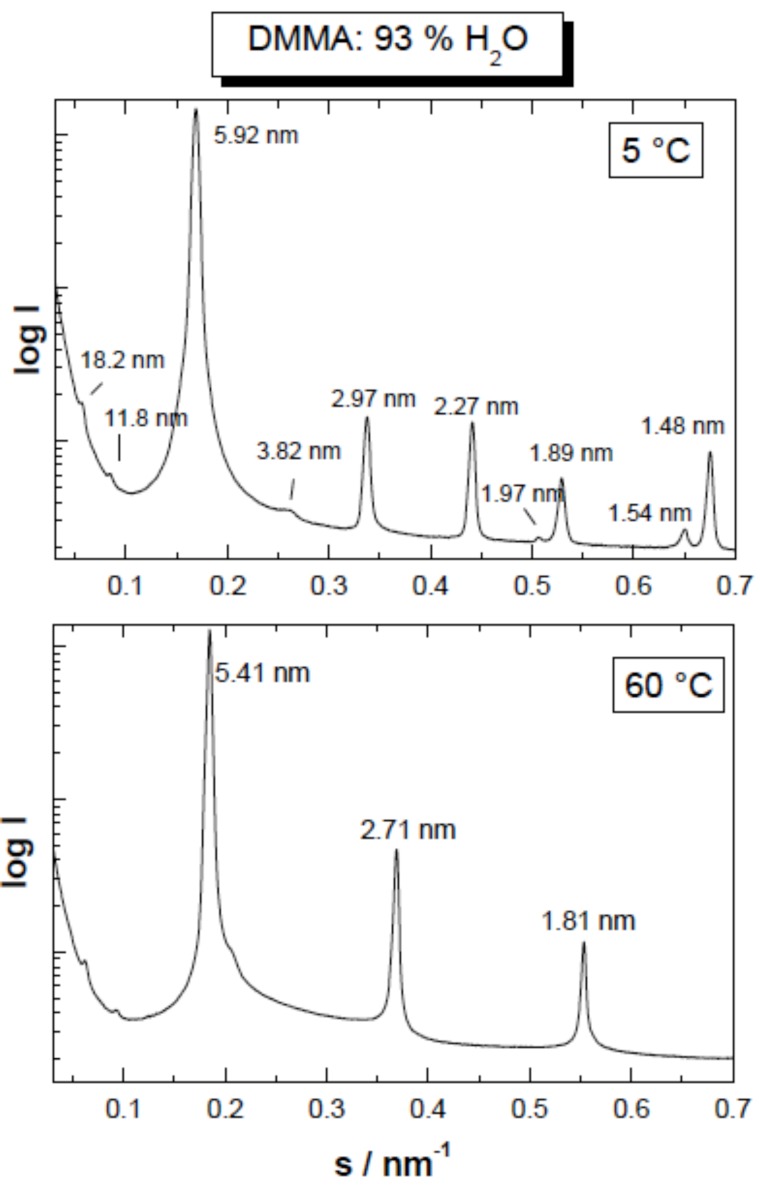
Small-angle X-ray scattering patterns of DMMA at 93 % water content and two temperatures 5 °C (top) and 60 °C (bottom). The logarithm of the scattering intensity is plotted *versus* the scattering vactor s (=1/d, d= spacings of the reflections). From [40] with permission by Elsevier.

Also very highly ordered phases with crystalline symmetry are found in some cases, as illustrated for dimyristoyl-maltoside in Fig. (**20**). Here, at 5 °C reflections are observed at 5.92, 2.97, 1.97, and 1.54 nm, which would correspond to a multilamellar phase with the periodicity and the corresponding higher order reflections (top). The other reflections at 2.27, 1.89, and 1.48 nm are not correlated to the former, and are indicative of a higher symmetry than a multilamellar stack, for example a triclinic or an orthorhombic phase [90]. On heating, these crystalline phases disappear and give rise to a normal liquid-crystalline phase at 60 °C (Fig. **20**, bottom). 

The wide-angle scattering range comprises the packing of the acyl chains. Normally the acyl chains of glycolipids are packed hexagonally, i.e. in a geometry described above for the long-range order systems. However, more ordered crystalline structures also occur, which may give rise to various unrelated sharp reflections. 

## 5 THERMOTROPIC AND LYOTROPIC PROPER-TIES OF GYLCOLIPIDS

In the last decade the number of papers, written about glycolipids and their physico-chemical properties, especially their thermotropic and lyotropic properties has increased (see references presented in this paper). Glycolipids have a particular relevance as liquid-crystalline materials [7, 91].

Hato and coworkers have observed a stereochemical dependent self-assembly of the hydrated lipid for the glycolipid 1,3-di-O-dodecyl-2-(β-maltoheptosyl)glycerol [92]. The reason for such behaviour is due to differences in the head group conformation of the cello-oligossacharide (see also Hato and coworkers 1996 [78], 1998 [92], 2001 [93]).

The thermotropic and lyotropic phase behaviour of diastereomer glycoglycerolipids containing a single hexopyranoside or pentopyranoside head group have been extensively studied by Mannock and McElhaney (2004) [65]. Using DSC and X-ray for the investigation of the physico-chemical properties of certain diastereomer glycoglycerolipids, it was suggested that both - head group and interfacial hydration - determine the lyotropic as well as the mesomorphic phase properties. It was observed that for glycoglycerolipids of a given chain length, the interactions between the head group interface and water molecules determine whether or not a highly ordered, lamellar crystalline phase is formed. Furthermore, the number of such phases and their rate of formation and, in some cases, the nature of the molecular packing of those phases is determined by these interactions [64, 65]. In the liquid-crystalline phases, the hydrocarbon chains determine the area per molecule in the lamellar liquid-crystalline phase, but it is the cross-sectional area of the hydrated head group and the penetration of water into the interface which determines the nature of the non-lamellar phases, probably through small changes in interfacial geometry as the lateral stresses in the head group region increase [65].

Amidic glycolipids with saccharide head groups and their physical properties as well as packaging states were presented by Takeoka *et al.,* (1998) [94]. Using the reactivity of saccharide against Concanavalin A, they were able to show that the reactivity of those saccharide glycolipids showing high reactivity probably was related to the loose packing of the saccharide moiety [94].

The analysis of the physico-chemical properties of various glycolipids are now available, for example: synthetic phytanyl-chained glycolipid/water systems [95], glycolipids with oligolactose head groups [9, 96], dialkyl glycolipids [46, 97], alkylpolyglacosides [93] or chiral glucose-derived surfactants [98], long-chain alkylmaltosides [99] or glycopyranosides [100], the in-plane miscibility and mixed bilayer microstructure formation of cationic glycolipids and zwitterionic phospholipids [101], disubstituted sucrose esters [102], monoacylates maltose glycolipids [103], dioleoyl glycolipids with mono- and disaccharide head groups [104], monoacylated amide-linked disaccharide glycolipids [105], amphiphilic branched chain glycolipids [106] or cellobiose alkanoates [107].

The phase transitions of glycolipids are often kinetically hindered. Rapp and colleagues have used X-ray to inves-tigate the glycolipid phase kinetics [108, 109], which is quite complex depending on the history of the sample preparation as well as storage conditions and the presence of additional components.

Certain glycolipids have very unspecific properties. For example, antiferroelectric ordering, which was observed for some glycolipids in bent-core liquid-crystals [110].

In the last years, efforts were undertaken to model and simulate the thermotropic and lyotropic alkyl glycoside bilayers [111] in order to understand the molecular principles for the formation of specific phases.

Various excellent reviews are available which deal with the self-assembly of glycolipids (for examples: Corti *et al.,* 2007 [112], Dumoulin *et al.,* 2002 [113]).

## 6 BIOLOGICAL AND PHYSIOLOGICAL IMPLICA-TIONS

Generally speaking, sphingolipids with short or longer sugar chains play an important role in, for example, the formation of membrane domains, which are involved in membrane-associated events such as cell signalling, adhesion, and protein sorting. These domains, called rafts, are frequently consisting of long-chain sugar sphingolipids called gangliosides [114, 115]. Of course, not all different glycolipid components of cellular membranes mix ideally with the usual phospholipids and not even with chemically similar compounds. Thus, Maggio studied the interaction mechanisms in Langmuir monolayer systems of ceramides, glycosphingolipids, and gangliosides [116]. He found different basic tendencies for the glycosphingolipids to molecularly mix and demix, and to be preferentially located in liquid-expanded or liquid-condensed states. A general presentation of the behaviour of glycosphinolipids in complex biomembranes was published by Prinetti *et al.,* (2009) [117], and details of the complex interplays between different glycosphingolipids and the tensions in lateral and transversal planes caused by them were presented by Maggio and others [118-120].

Five years ago, Goodby *et al.,* 2007 [121] have reviewed the thermotropic liquid-crystalline glycolipids. In their introductory part of the paper, they ask whether liquid-crystalline properties of the materials of living systems are important and what is the relevance for biological structures, functions, diseases and treatments [121]. Goodby and coworkers show evident that these materials play a certain role in biology. They mention that over the last years, the observed lyotropic, and often thermotropic liquid-crystallinity of many biological materials that are linked to key biological functionalities might be more than just a curious coincidence. There are different aspects of glycolipids and their properties as liquid-crystalline compounds that are crucial for biological systems. For example, Vill has analysed and discussed the stereochemistry of glycolipids and linked this property to membrane functions [122].

As amphiphiles glycolipids self-assemble forming different structures and phases, so called “soft-matters”, which combine the properties of structure and flexibility. Flexibility and adaptation (re-organisation and the formation of various phases) are key aspects for biological systems [123]. Furthermore, the water response of certain glycolipids and the formation of lyotropic phases are also of high relevance. Depending on the chemical nature of the glycolipid, they are also able to form stable micelles and vesicles [105, 124-126].

The analysis of biological cells shows that a large variety of glycolipids can be isolated. Beside the phospholipids, glycolipids are important components of cell membranes. In certain cell membranes the concentration of glycolipids is much higher [127]. For example, the amounts of glycolipids like cerebrosides, globosides or gangliosides are of higher concentration in nerve cell membranes compared to other lipids. These specific glycolipids are related to the appearance of certain diseases. An increase in cerebrosides induces in humans the manifestation of diseases like Gaucher’s or Krabbe’s disease Further examples are listed in Table **2**. 

Glycolipids are also related to the formation of inhomo-geneities, i.e. the formation of domains in biological membranes. The role of these domains is still being controversially discussed [128-130].

Glycolipids are also highly present in bacteria of the extremophile group [127, 131]. 

Certain glycolipids are responsible for anti-infective resistance and anti-bacterial properties. Lotfabad *et al.,* (2013) have evaluated the antibacterial capability of rhamnolipids (MR01 and MASH1) against several specified microor-ganisms [132]. They observed remarkable inhibitory effect against Gram-positive bacteria, but none of the two tested glycolipids MR01 and MASH1 showed significant effects on Gram-negative bacteria growth inhibition [132, 133].

One example of a glycolipid with anti-infective resistance, is the cell wall of mycobacteria which contains highly specifically branched lipids based on arabinogalactan. These glycolipids termed as mycolic acids are the molecular reason for the high resistance of mycobacteria against most traditional anti-infectives [134]. 

Various glycolipids are known for having certain physiological impacts. For example, the cord factor, 6,6’-dimycoclic ester of a,a-trehalose, is linked to a virulent strain of *tubercle bacilli* [135, 136]. Furthermore, the cord factor has immunostimulatory properties and shows antitumour activity [137]. In an aqueous medium it also causes bacteria to form cords (Goodby *et al.,* 2007 [121] and references cited therein, Almog and Mannella 1996 [130]). 

Related to liquid-crystalline materials, the cord factor exhibits a thermotrope-induced cubic phase [130, 137].

Saponins are a class of glycosides linked to steroids, steroidal alkaloids or triterpenes found in a number of plants (e.g. *Agrostemma githago*, *Lonicera*, *Aesculus hippocasta-num*). Dissolved in water, saponins induce the formation of an alkaline solution. This is the reason for the name saponin, which is derived from the latin word *sapo*, meaning soap. Due to this property saponins are capable of hemolysing erythrocyte cells. The bioactivity of saponins has been described by Rao and Gurfinkel 2000 [138]. Taken *via* the peroral route, most saponins induce more or less toxicity. In case of peroral intoxication, activated-carbon should be given directly followed by a concrete treatment.

A therapeutically relevant example of this class of glycolipids is the cardio-active drug digitoxin. Additional examples of glycolipids and their derivatives as therapeutic agents can be found elsewhere (for a summary see Sakai and Koezuka 1999 [139], Moran *et al.,* 2009 [127]). As an example, for the therapeutic application of glycolipid (an analog of alpha-galactosylceramide with a truncated sphingosine chain) a synthetic glycolipid is tested for the treatment of multiple sclerosis [140]. Oral administration of this glycolipid induces Th2 bias of autoimmune T cells *via* the production of interleukin 4 by NKT cells, leading to the suppression of encephalomyelitis [140].

Different glycolipids are linked to immunological activity. For example, mycobacterial trehalose-containing glycolipids show immunomodulatory activity on human CD4^+^ and CD8^+^ T-cells [141]. Immunomodulation by zwitterionic polysaccharides is reviewed by McLoughlin and Kasper (2009) [142]. Reed *et al.,* (2004) [143] have presented a glycolipid study related to a hypervirulent tuberculosis strain that inhibits the innate immune response. The text by Apicella and Jennings (2009) [144] deals with the phase variation of bacterial surface glycosylated molecules in immune evasion. The use of glycomimetics as inhibitors in anti-infection therapy has been recently described by Kiefel (2009) [145] and applications of bacterial polysaccharide vaccines have been presented by Jennings and Pon (2009) [146].

The paper by Savage *et al.* [147] focuses on glycolipids for natural killer T cells [147] and Tsuij (2006) [148] described different glycolipids and phospholipids that function as natural CD1d-binding NKT cell ligands [149].

Recently, Takahashi *et al.,* (2012) observed an antioxidant effect of glycolipid biosurfactants of the class of mannosylerythritol. The results of their study show that the investigated glycolipid has protective effects against water peroxide induced oxidation of human skin fibroblasts [150].

It also states that certain glycolipids are not observed in mammals [150]. In the monograph by Moran *et al.,* 2009 [127] entitled “microbial glycobiology”, a number of examples can be found which will not be considered here.

Glycolipids are also tested as drug delivery systems. Bogdanneko *et al.,* (2005) [151] have used a lactose containing glycolipid in gene therapy for the targeted DNA delivery. Glycerate surfactants are tested as sustained release systems for drug delivery [152] (Boyd *et al.,* 2006).

Liquid-crystalline nanoparticulate systems have also been described and tested as drug delivery systems [153].

Also in the field of cosmetic applications some glycolipid biosurfactants are being evaluated [154-156]. Yamamoto *et al.,* (2012) evaluated the property of various mannosy-lerythritol lipids on the moisturising effect (water retention property) on human skin [157]. The outcome of the study suggested that these glycolipids are likely to exhibit a high moisturizing action by assisting the barrier function of the skin. This effect was linked to the unique feature of mannosylerythritol derivatived as liquid-crystal forming compounds [157].

Sulfatide, sulphated glycolipids, were recently reviewed by Compostella *et al.,* [158]. These glycolipids form a class of relevant endogeneous acidic glycolipids found in mammalian membranes. Sulfatides are an important constituent of brain lipids. Furthermore, sulfatides are an essential lipid class found in the peripheral as well as the central nervous system, with concentrations of ca 5 mole-% of the total lipids in adult brain myelin.

The biological relevance of certain lipopolysaccharides, especially their structure-related activity was investigated intensively by Brandenburg and co-workers [3, 159]. As a general rule, lipopolysaccharides with a cone-shaped structure induce inflammation (agonistic), whereas lipopolysaccharides with a lamellar-shaped structure are less active or show no activity at all (antagonistic) (see Fig. **21A**).

These are just a few selected examples for the biological implications of glycolipids in biology. Fig. (**21B**) summarises the current knowledge about the relationship between the self-organisation and supra-molecular structure of various lipopolysaccharides with biological activity, more explicitly, endotoxicity [3]. For this class of glycolipids one can tentatively conclude that a more rectangular shape of the single LPS molecule show reduced or are inactive, whereas cone-shaped LPS molecules show increased endotoxicity [3, 159]. However, it is clear that a lot is currently unknown and a number of efforts are still necessary to get a better understanding with regards to the biological relevance of glycolipids. 

## Figures and Tables

**Fig. (4):**
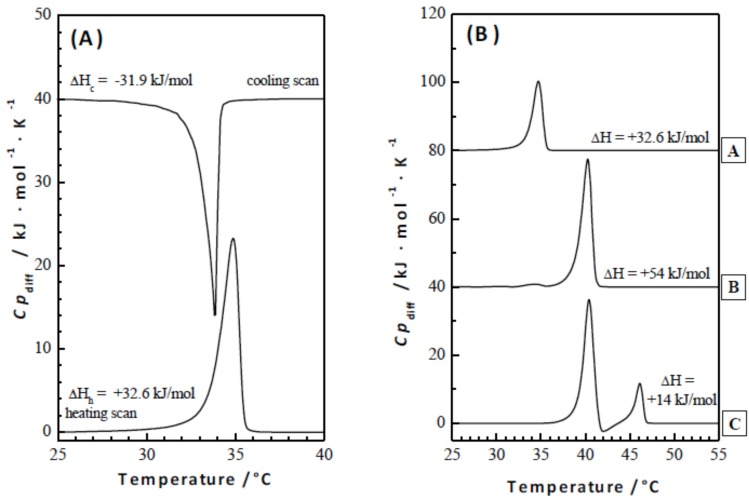
Heat capacity curves of DMMA (1,3-Di-O-Tetradecyl-2-O-[4’-O-(α-D-glucopyranosyl)-ß-D-glucopyranosyl]-sn-glycerol) in PBS (phosphate buffered saline) buffer at pH 7.4; scan-rate 1 K/min. (A) Heating and cooling scan of a freshly prepared dispersion. (B) Heating scan of [A] A freshly prepared sample, [B] Sample stored for 7 days at 4 °C, [C] Sample stored for 21 days at 4 °C. Adapted from [40].

**Fig. (5):**
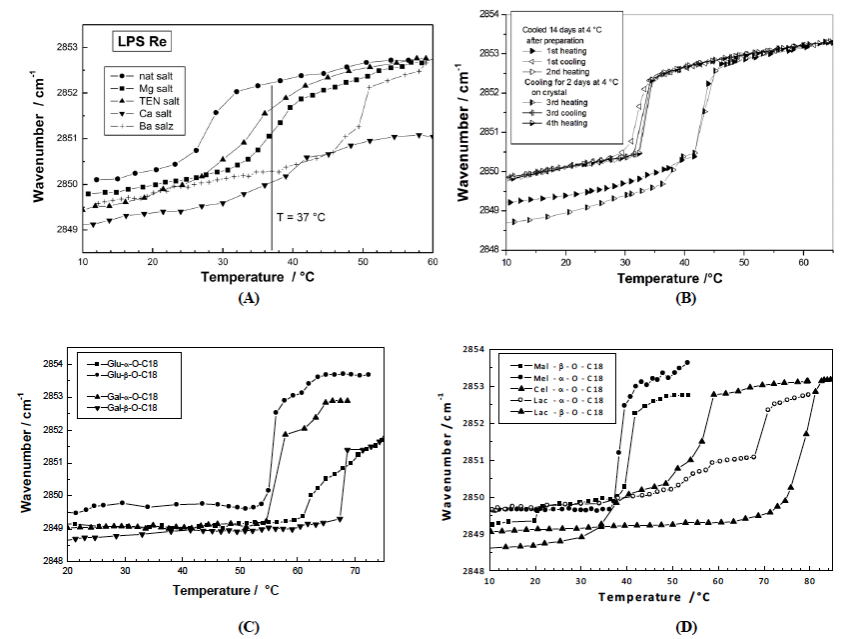
Phase transition of various glycolipids by infrared spectroscopy. The peak position of the symmetric stretching vibrational band of the methylene groups vs(CH2) is plotted versus temperature. (A) Different salt forms of lipopolysaccharide from deep rough mutant R595 from Salmonella minnesota. From [59] with permission by Elsevier. (B) 1,3-Di-O-tetradecyl-2-O-[4’-O-(α-D-glucopyranosyl)-β-Dglucopyranosyl]- sn-glycerol (DMMA). From [40] with permission by Elsevier. (C) Stearyl monosaccharides galacto and glucolipids Gal-α- OC18, Gal-β-OC18, Glu-α-OC18, and Glu-β-OC18. After [67] with permission by Elsevier. (D) Stearyl disaccharides, maltosides, melibiosides, cellubiosides, and lactosides in different linkages. From [67] with permission by Elsevier.

**Fig. (6):**
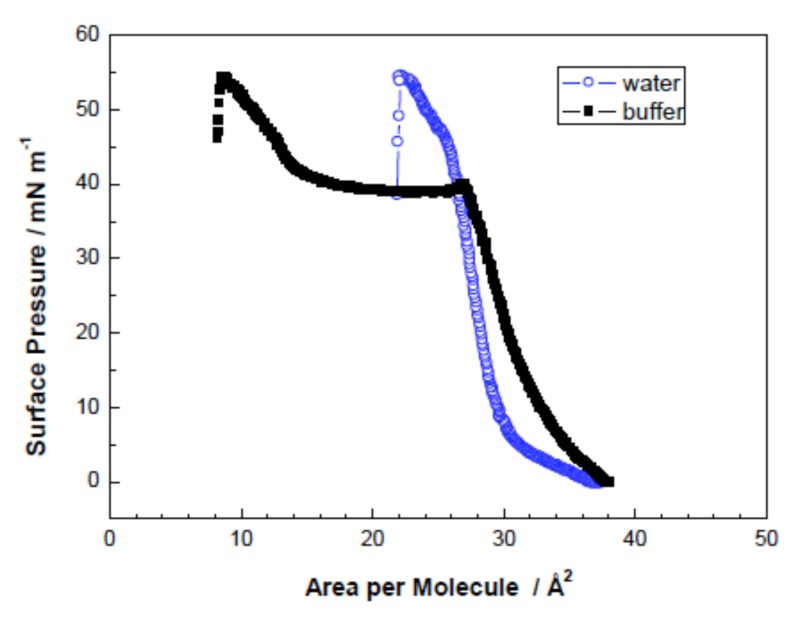
Surface pressure/area-isotherms of DMMA in water and 20 mM HEPES at 20 °C (Adapted from [40]).

**Fig. (7A1):**
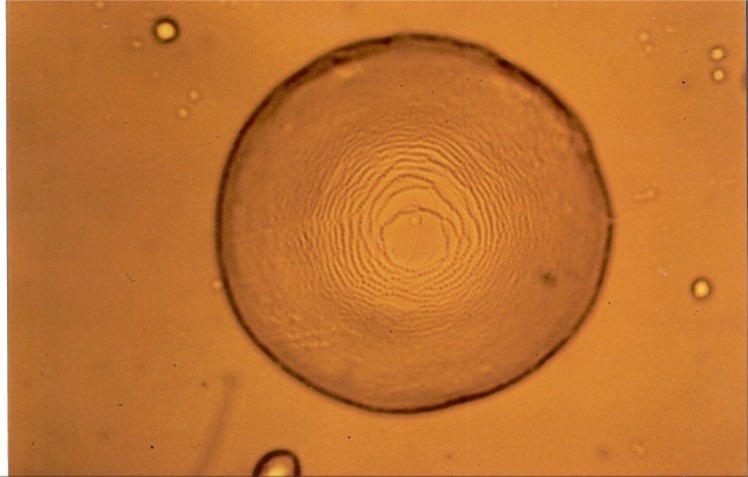
Smectic A “Stufentropfen” of glycolipid MalC12.

**Fig. (7A2):**
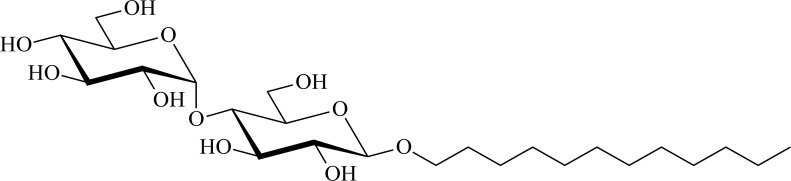
Chemical structure of the glycolipid MalC12.

**Fig. (7B1):**
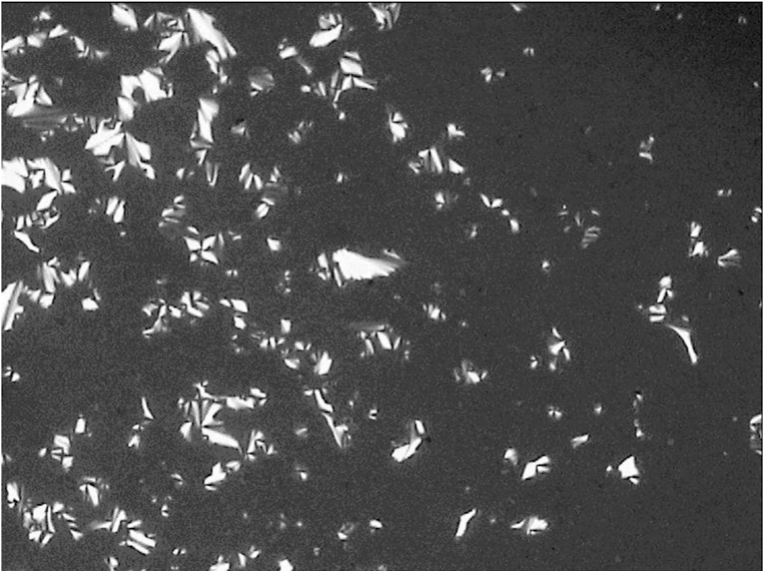
Smectic A fan-shaped texture of glycolipid GluC11:1w on cooling at 131.7° C.

**Fig. (7B2):**
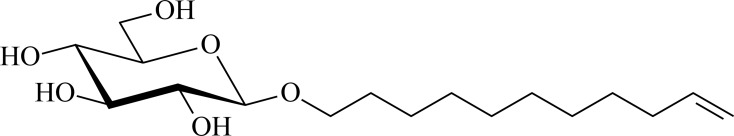
Chemical structure of the glycolipid GluC11:1w .

**Fig. (7C1):**
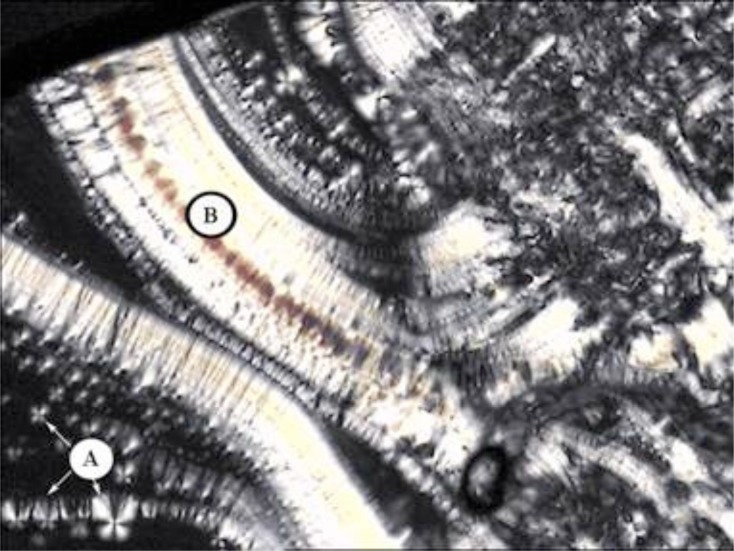
Smectic A with maltese crosses (A) and pearlchains (B) of glycolipid GalC11.

**Fig. (7C2):**
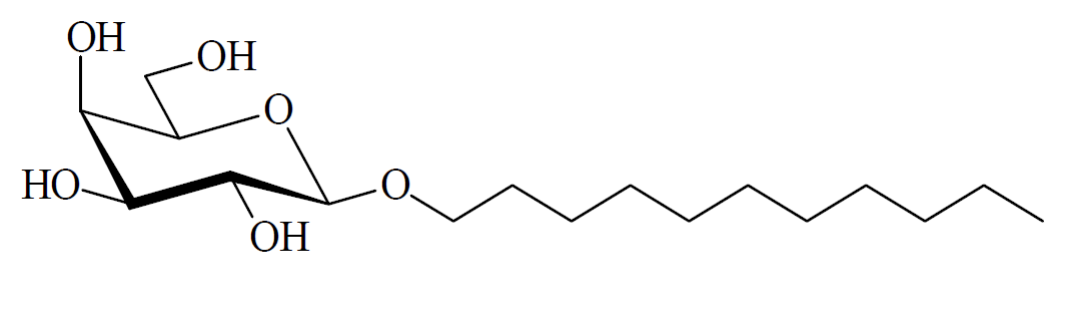
Chemical structure of the glycolipid GalC11.

**Fig. (7):**
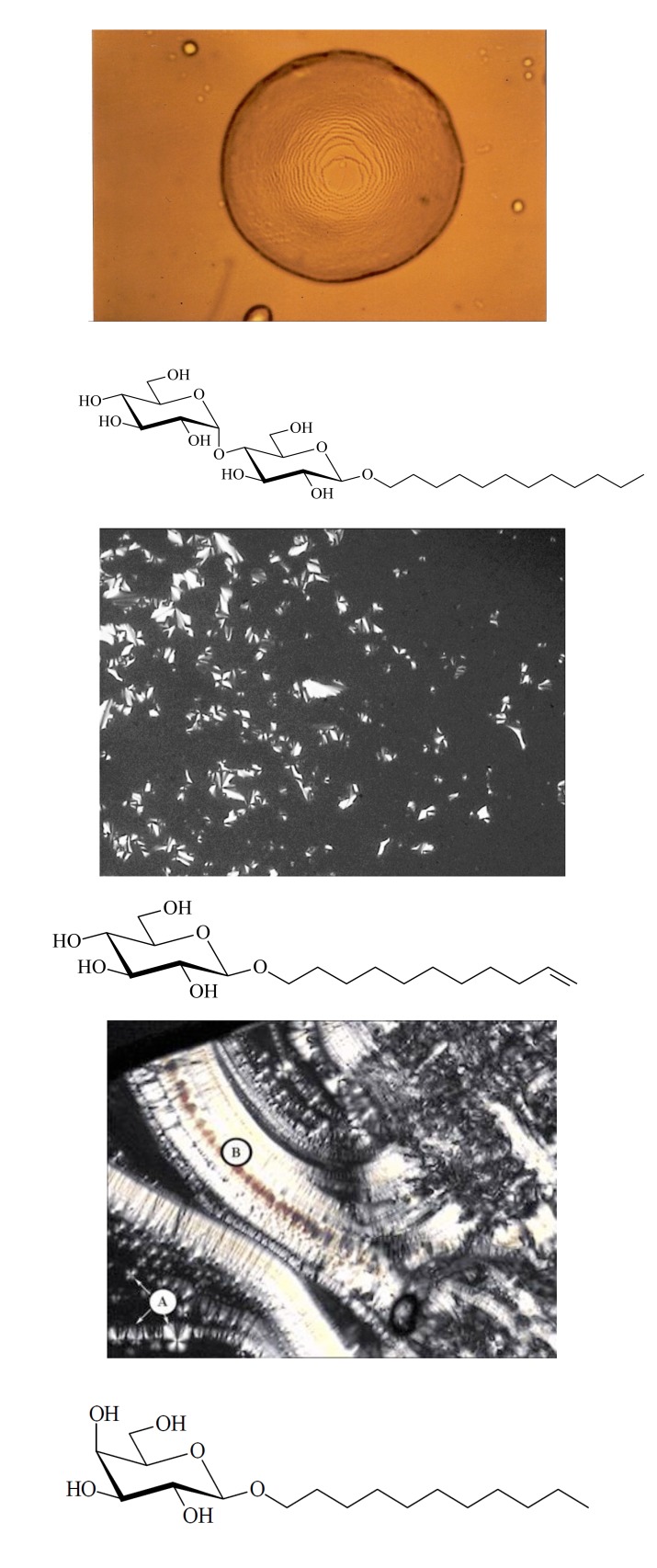
Pictures obtained with polarization microscopy.

**Fig. (8A1):**
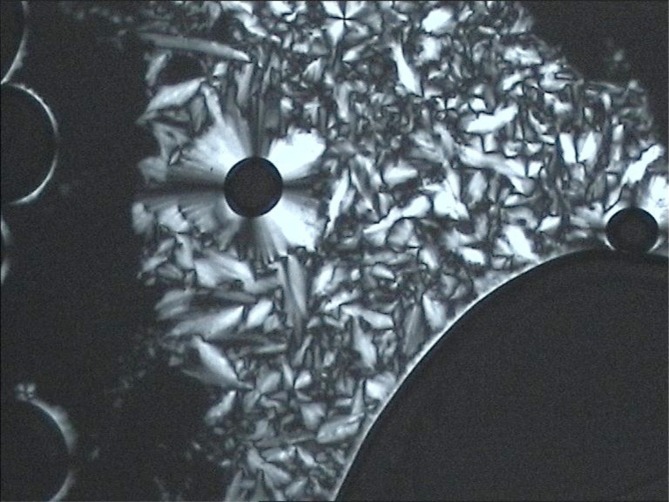
Texture of a columnar phase of glycolipid MelC12N3.

**Fig. (8A2):**
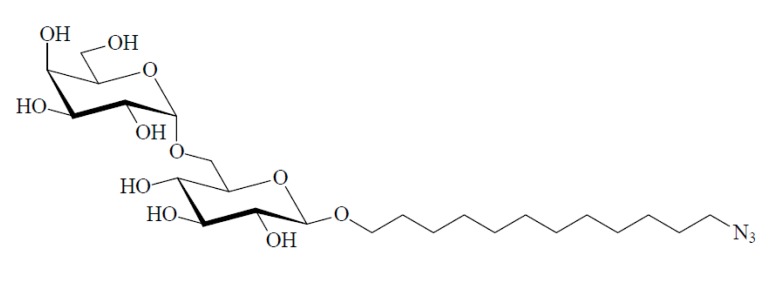
Chemical structure of the glycolipid MelC12N3.

**Fig. (8B1):**
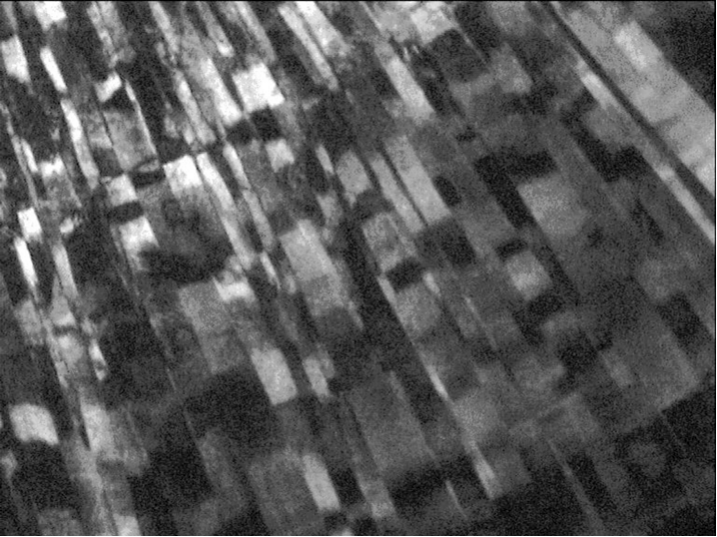
Unusual texture for a columnar phase of glycolipid MelC11, contact preparation in water.

**Fig. (8B2):**
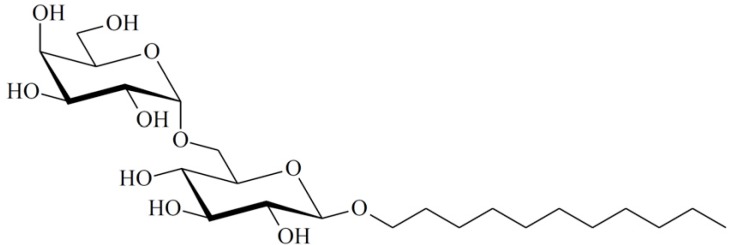
Chemical structure of the glycolipid MelC11.

**Fig. (8C1):**
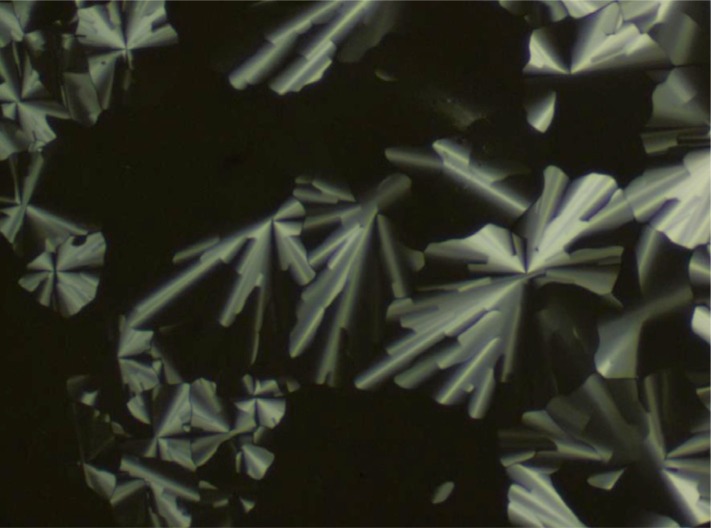
(C-1). Columnar Dho (hexagonal columnar ordered) phase of CellC12(8) [107].

**Fig. (8C2):**
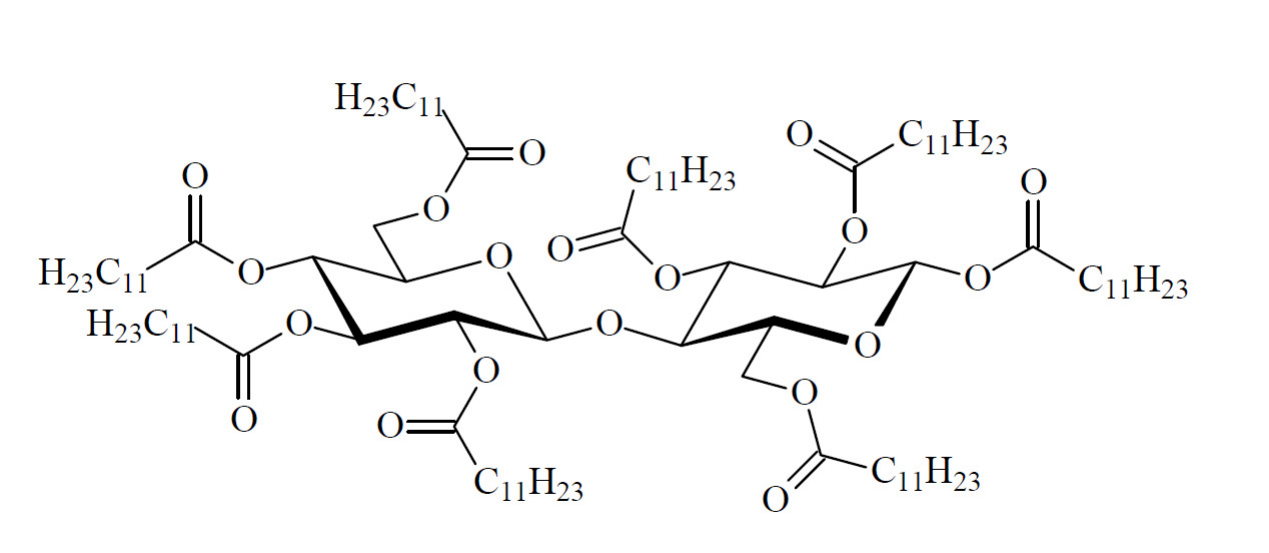
Chemical structure of the glycolipid CellC12(8).

**Fig. (8):**
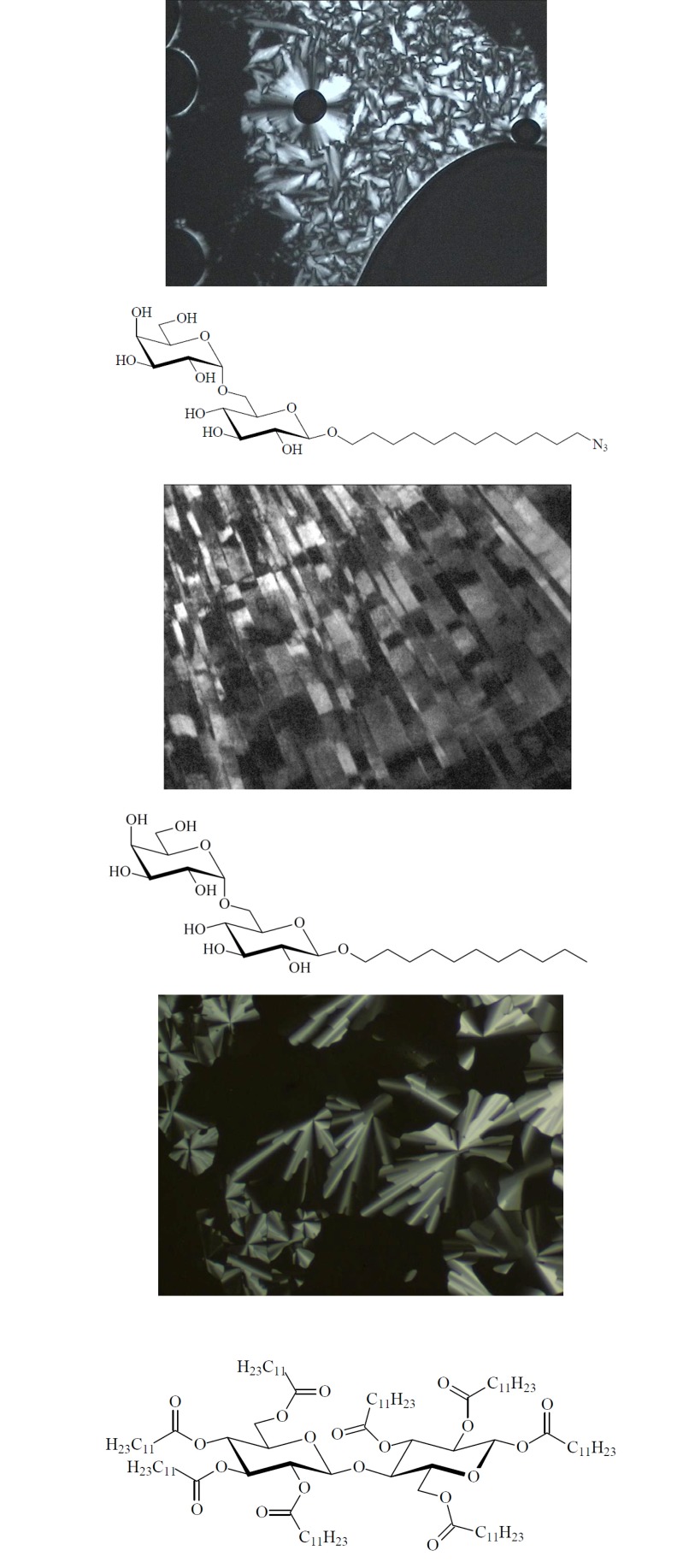
Pictures obtained with polarization microscopy.

**Fig. (9A1):**
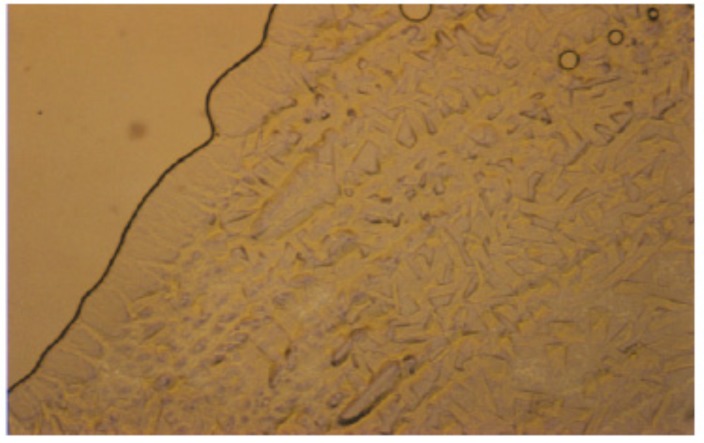
Unusual texture for a cubic phase of the 1:1- glycolipid mixture of Cel-β-glycero dioleoyl and Glu-β-glycero dioleoyl, contact preparation in water.

**Fig. (9A2):**
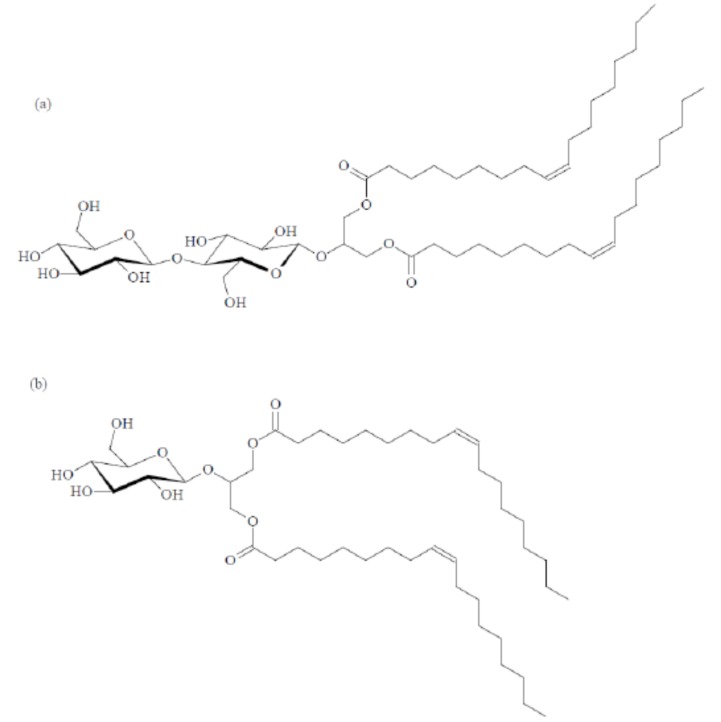
Chemical structures of the glycolipids Cel-β-glycero dioleoyl (a) and Glu-β-glycero dioleoyl (b).

**Fig. (9B1):**
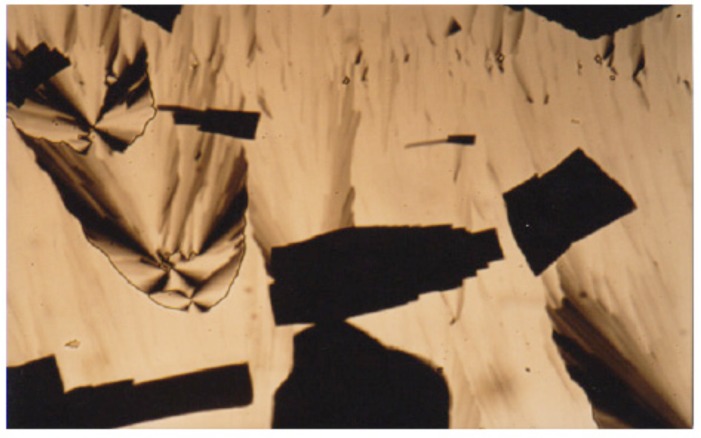
Texture of a cubic phase of the Glu-β-glycero didecanoyl.

**Fig. (9B2):**
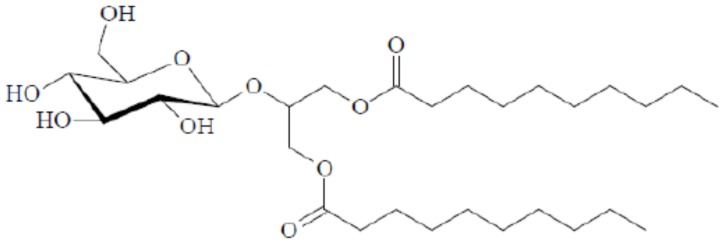
Chemical structure of the glycolipid Glu-β-glycero didecanoyl.

**Fig. (9):**
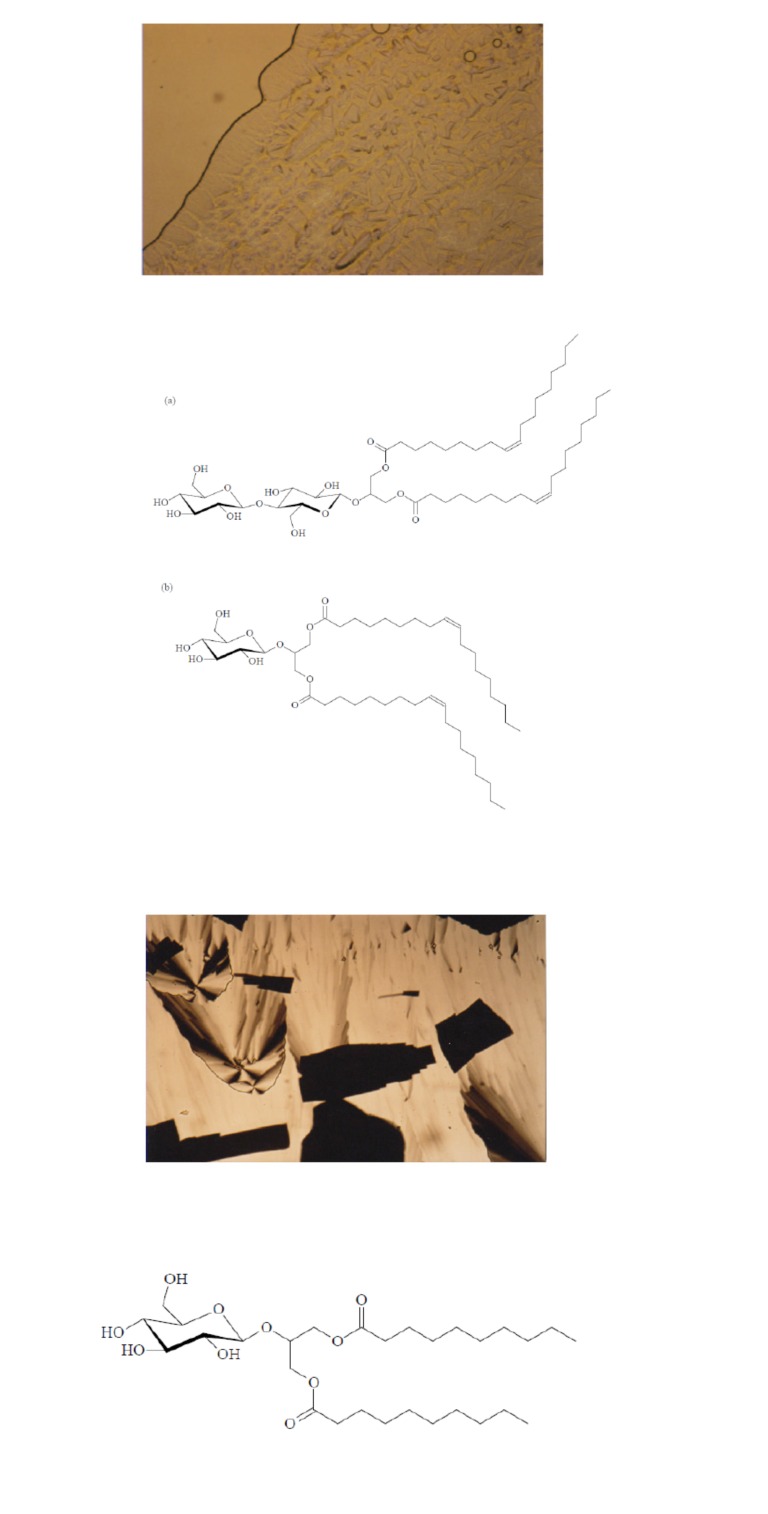
Pictures obtained with polarization microscopy.

**Fig. (10A1):**
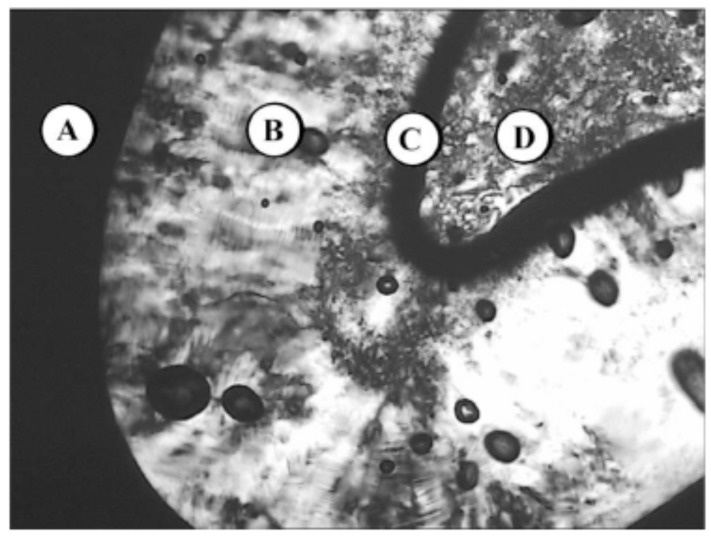
Lyotropic phase behaviour of glycolipid MalC12N3 - (A) water, (B) columnar HI, (C) bicontinous cubic VI, (D) pure compound (SmA).

**Fig. (10A2):**
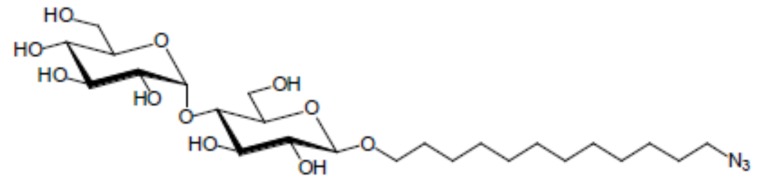
Chemical structure of the glycolipid MalC12N3.

**Fig. (10B1):**
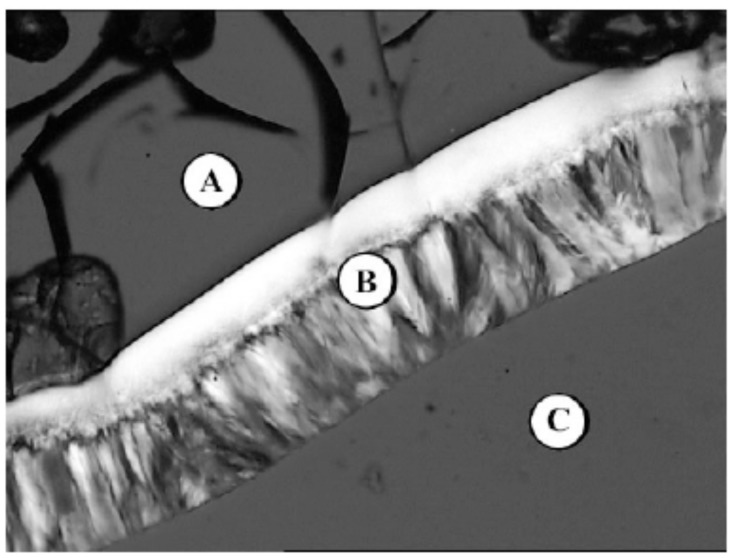
Lyotropic phase behaviour of the glycolipid GenC12N3: (A) pure compound (glass), (B) hexagonal HI, (C) water.

**Fig. (10B2):**
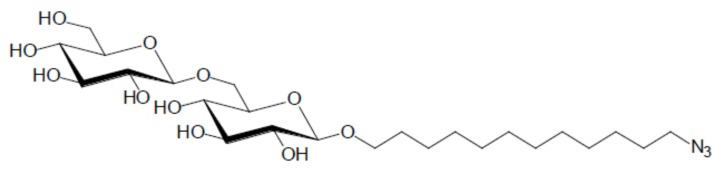
Chemical structure of the glycolipid GenC12N3.

**Fig. (10C1):**
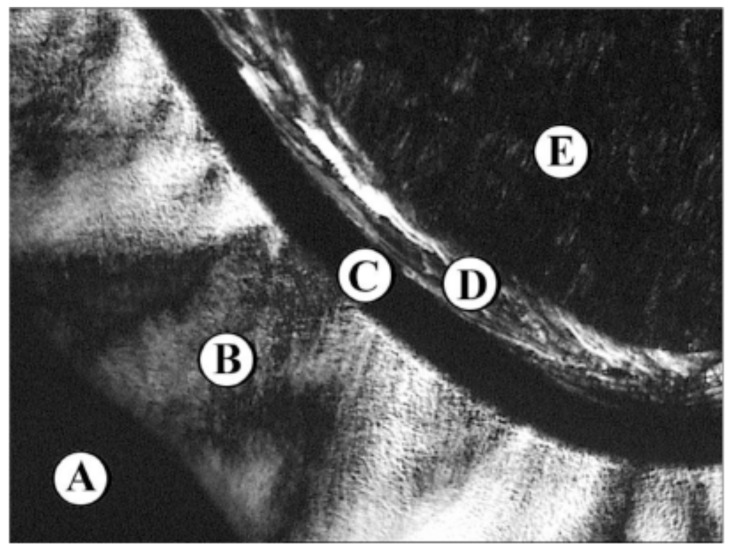
Lyotropic phase behaviour of the glycolipid Lac- CerPiv: (A) Water, (B) lamellar La, (C) bicontinous cubic V1, (D) hexagonal HI, (E) pure compound SmA.

**Fig. (10C2):**
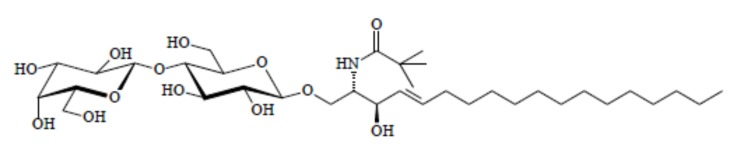
Chemical structure of the glycolipid LacCerPiv.

**Fig. (10):**
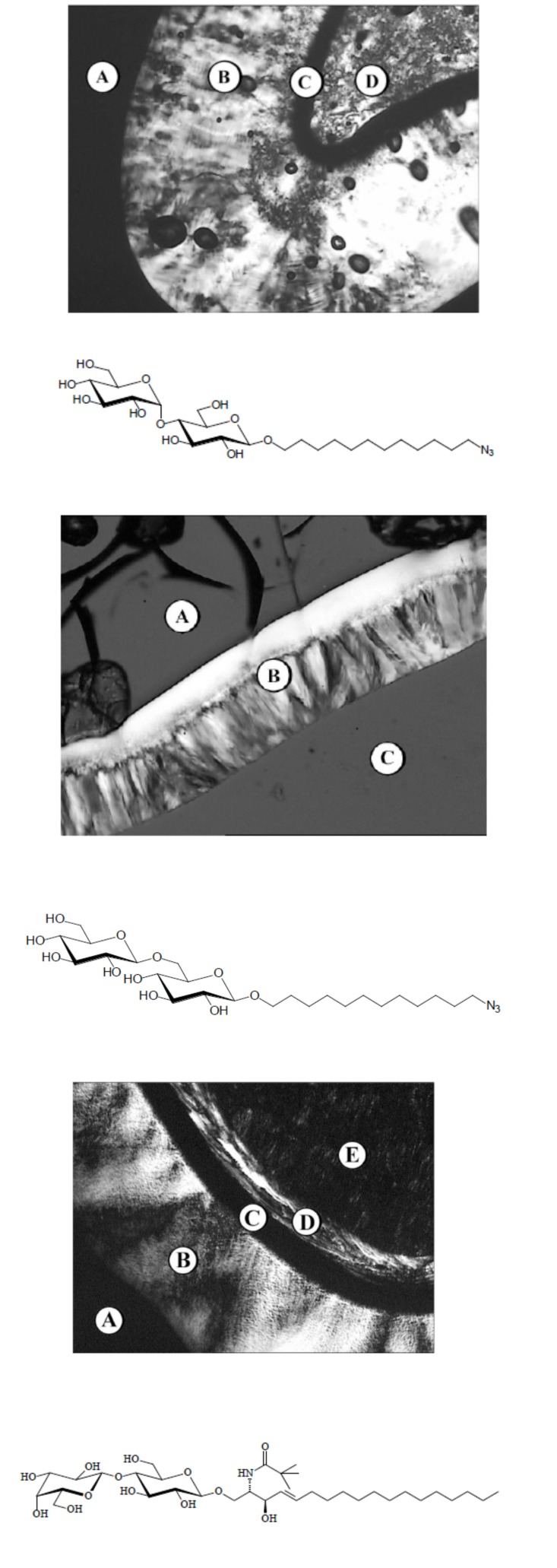
Pictures obtained with polarization microscopy.

**Fig. (11A1):**
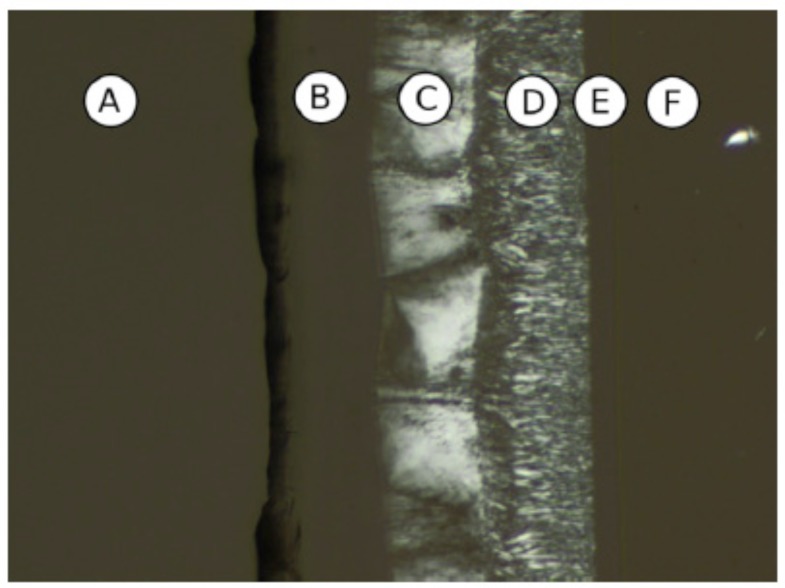
Contact preparation of MalOC3OC12 in water. (A) Water, (B) cub, (C) Col, (D) Col, (E) cub, (F) pure compound (SmA).

**Fig. (11A2):**
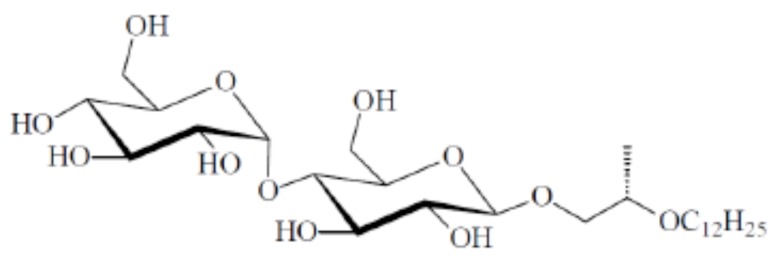
Chemical structure of the glycolipid MalOC3OC12.

**Fig. (13):**
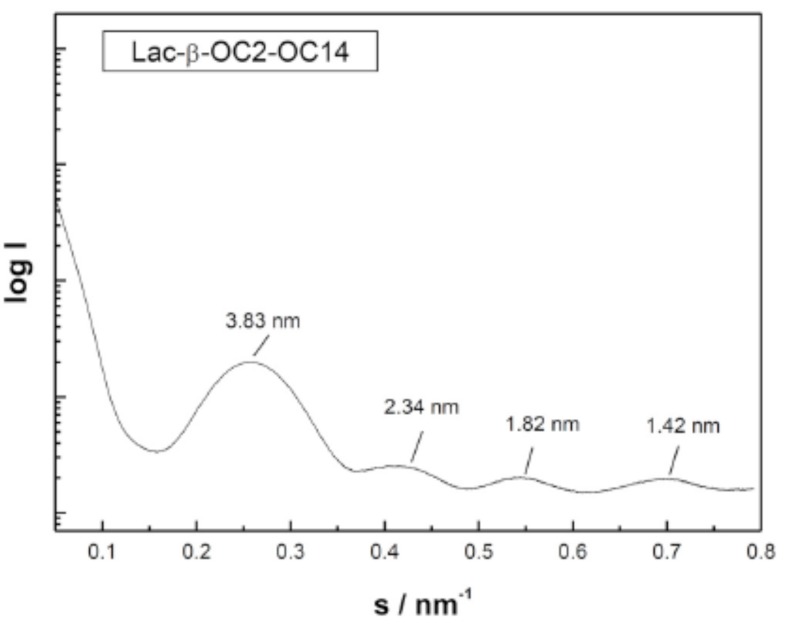
Small-angle X-ray scattering (SAXS) patterns of a monoacylated lactoside Lac-β-OC2-OC14 . The reflections are characteristic for the existence of a micellar phase. The logarithm of the scattering intensity is plotted versus the scattering vector s (=1/d, d = spacings of the reflections).

**Fig. (14):**
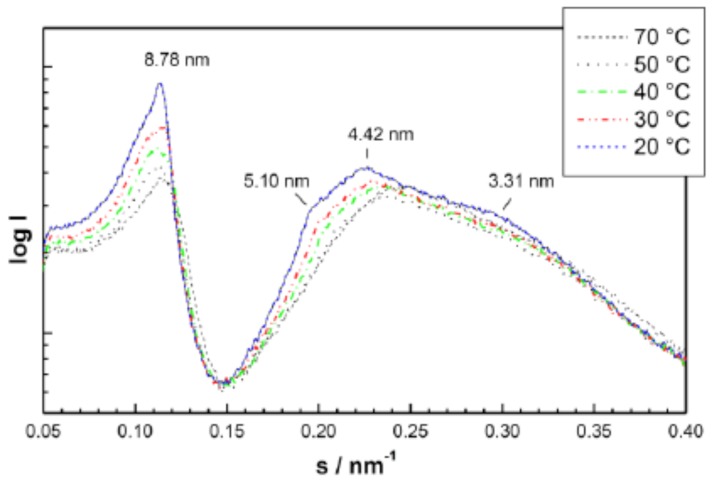
Small-angle X-ray scattering (SAXS) patterns of a triacylated lipid A compound OM-174. The reflections are characteristic for the existence of the direct hexagonal HI –phase. The logarithm of the scattering intensity is plotted versus the scattering vector s (=1/d, d = spacings of the reflections). After [62].

**Fig. (15):**
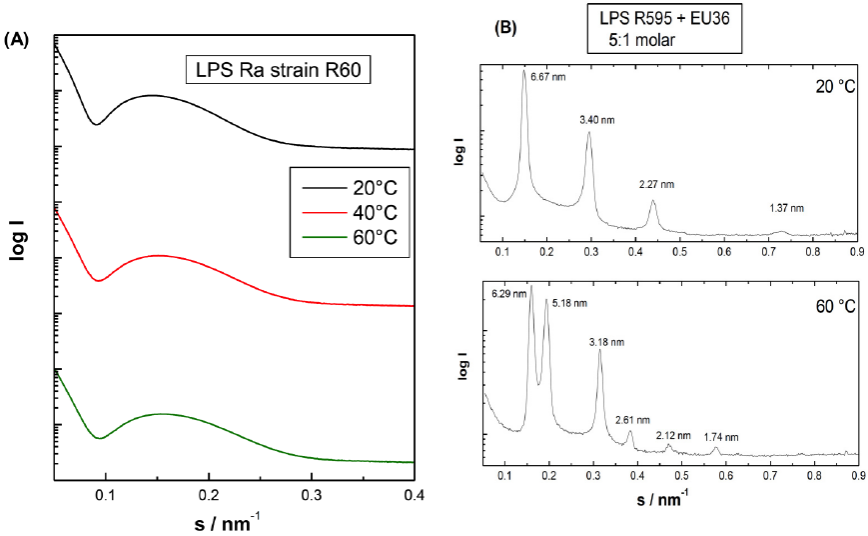
Small-angle X-ray scattering (SAXS) patterns of lipopolysaccharide Ra from *Salmonella minnesota* strain R60. The broad scattering profiles characteristic for the existence of an extended bilayer (form factor). Additionally, the small scattering peaks in the low srange could be indicative for the existence of a cubic structure. The logarithm of the scattering intensity is plotted versus the scattering vector s (=1/d, d = spacings of the reflections). Own unpublished data. (B). Small-angle X-ray scattering (SAXS) patterns of lipopolysaccharide Re from *Salmonella minnesota* strain R595 in the presence of an antimicrobial peptide EU#36. The sharp reflection in equidistant ratios are characteristic for the existence of a multilamellar aggregate. The logarithm of the scattering intensity is plotted versus the scattering vector s (=1/d, d = spacings of the reflections). Own unpublished data.

**Fig. (16):**
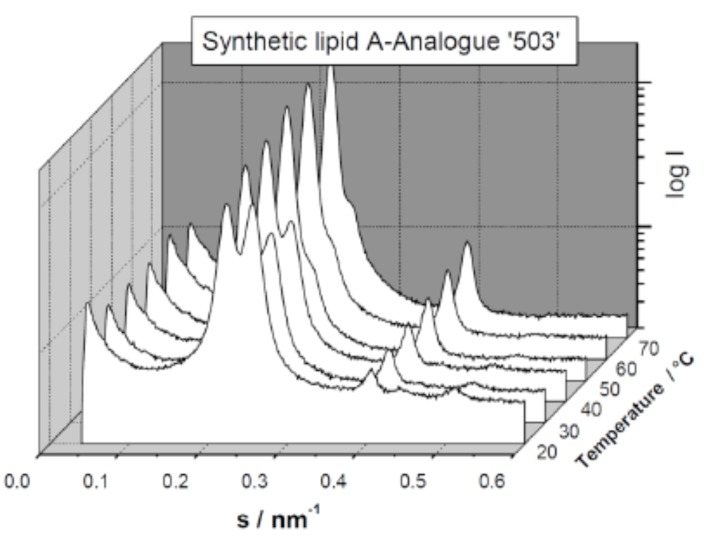
Small-angle X-ray scattering (SAXS) patterns of synthetic dephospho lipid A compound ‘503’. The sharp reflection in equidistant ratios are characteristic for the existence of a multilamellar aggregate. The logarithm of the scattering intensity is plotted versus the scattering vector s (=1/d, d = spacings of the reflections). Own unpublished data. Compound ‘503‘ was kindly provided by S. Kusumoto, University of Osaka.

**Fig. (17):**
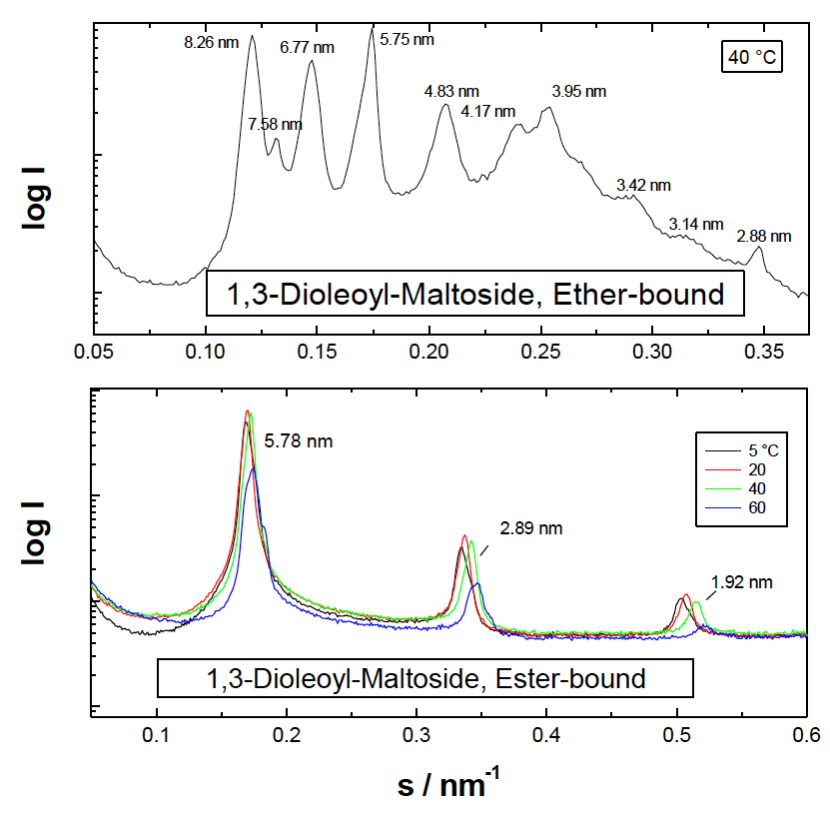
Small-angle X-ray scattering patterns of the diacylated monosaccharides dioleoyl-glucoside and –galactoside. The logarithm of the scattering intensity is plotted *versus* the scattering vector s (=1/d, d = spacings of the reflections). From [103] with permission by Elsevier.

**Fig. (18A):**
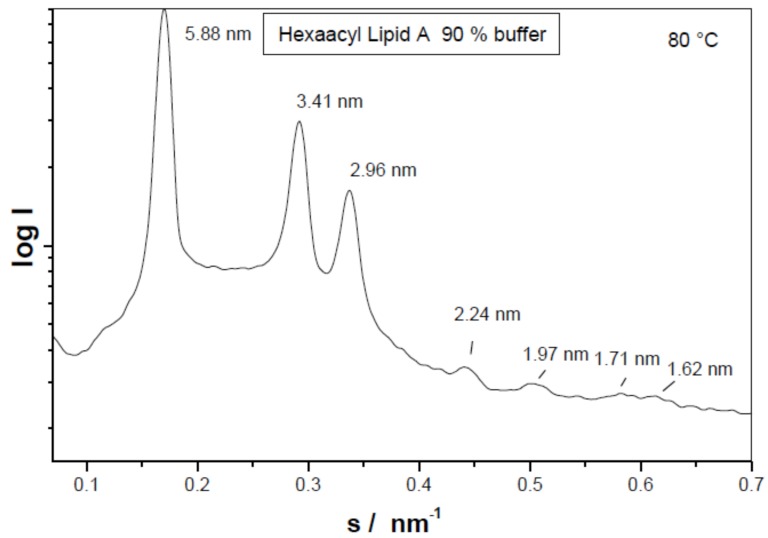
Small-angle X-ray scattering patterns of highly purified lipid A isolated from *Escherichia coli* F515 at 90 % water content and 80 °C. The logarithm of the scattering intensity is plotted versus the scattering vector s (=1/d, d = spacings of the reflections). The lipid A was kindly provided by U. Zähringer, Forschungszentrum Borstel. Own unpublished data

**Fig. (18B):**
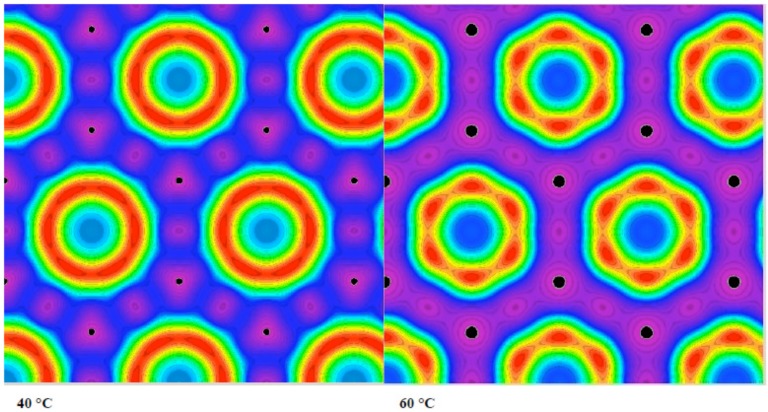
Electron density distributions of the H_II_ phase of hydrated at 40 and 60 °C.

**Fig. (19):**
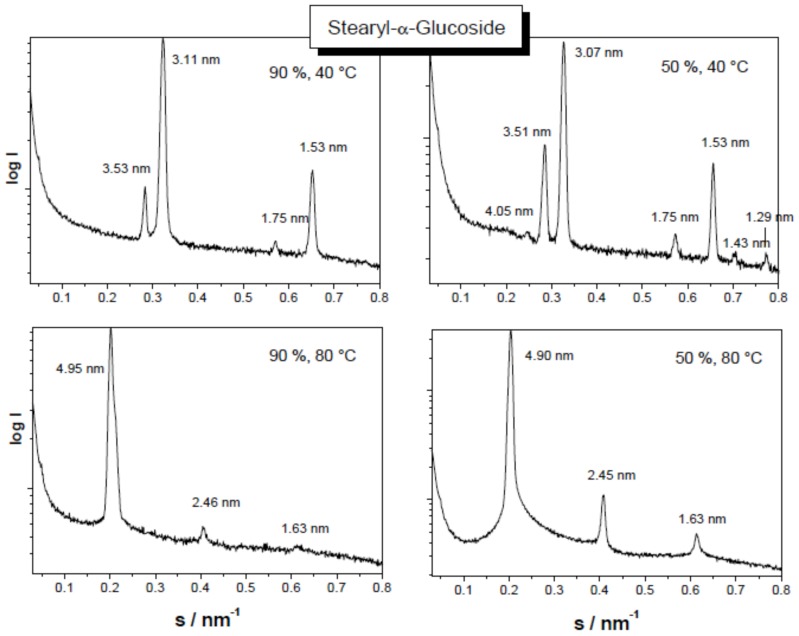
Small-angle X-ray scattering patterns of stearyl glucoside at 90 % (left spectra) and 40 % (right spectra) water content, and at 40 °C (top spectra) and 80 °C (bottom spectra). The logarithm of the scattering intensity is plotted *versus* the scattering vector s (=1/d, d = spacings of the reflections). From Vill *et al*., Chem. Phys. Lipids 104, 75-91 (2000), with permission by Elsevier.

**Fig. (21A):**
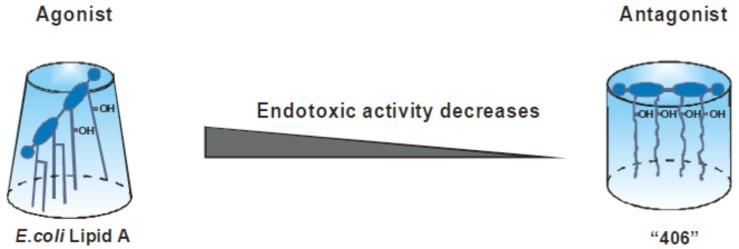
The generalised endotoxic principle. It should be noted that this conformational concept is valid also for non-LPS structures, such as the EISAI compound ER803022, a phospholipid with 6 acyl chains and two phosphate groups with a serine-like backbone.

**Fig. (21B):**
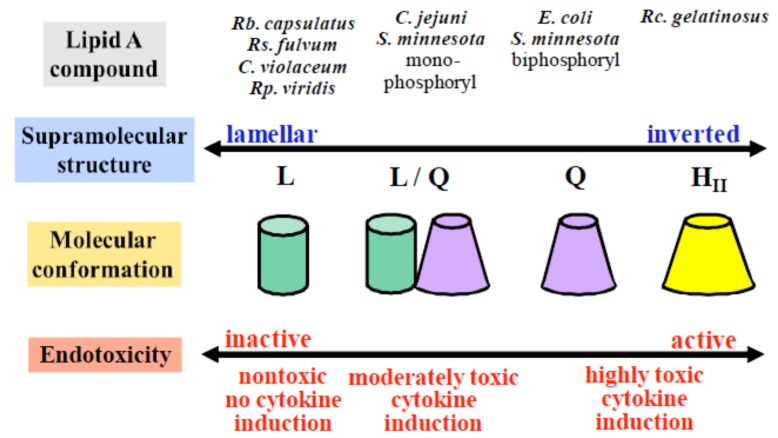
Correlation between supra-molecular lipopolysaccharide (LPS) structure and bioactivity. Adapted from [83].

**Table:** A. Thermotropic Properties.

Glycolipid	Observed Thermotropic Phases				
Mal-β-C14	Cr	107	SmA	264	I
Mal-β-C18	Cr	106	SmA	274	I
Mal-β-EA14	Cr	102	SmA	165	I
Mal-β-AB14	g	?	SmA	240	I

**Table:** B. Lyotropic Properties.

Glycolipid	Lyotropic Phases			
Mal-β-C14		Lα		H1
Mal-β-C18		Lα		H1
Mal-β-EA14		Lα*		
Mal-β-AB14		Lα	V1	H1
	Pure lipid			100% water

* = Myelin figures were formed in the contact preparation with water.

**Table 2.** Connection of Specific Glycolipids to the Expression of Diseases

Glycolipid	Connected Disease
Globoside	Fabry
Sphingomyelin	Nieman-Pick
Ceramide	Fabry
Ganglioside (GM2)	Tay-Sachs
Glucose cerebroside	Gaucher

## ABBREVIATIONS

Col: = Columnar phase
Cub: = Cubic phase
H_1_: = Hexagonal phase (lyotropic columnar) = H_I_
H_2_: = Inverted hexagonal phase (lyotropic columnar) = H_II_
I_1_: = Discontinuous cubic phase
I_2_: = Inverted discontinuous cubic phase
LPS: = Lipopolysaccharide
L_1_: = Micellar phase
L_2_: = Inverted micellar phase
L_α_: = Lamellar phase
L_C_: = Liquid-crystalline
SmA: = Smectic A phase
SmB: = Smectic B phase
SmC: = Smectic C phase
V_1_: = Bicontinous cubic phase = V_I_
V_2_: = Inverted bicontinous cubic phase = V_II_
